# Supplementary material for: Friends with malefit. The effects of keeping dogs and cats, sustaining animal-related injuries and Toxoplasma infection on health and quality of life
Source: PLoS One. 2019 Nov 22;14(11):e0221988. doi: 10.1371/journal.pone.0221988 (PMC6874301; doi:10.1371/journal.pone.0221988)
Supplement: S2 Fig — The boxes, spreads, upper numbers and lower numbers show standard errors, standard deviations, numbers of subjects in particular category and p-values of two-sided t-tests, respectively. (PDF) [file pone.0221988.s002.pdf]

Fig. S2 Effect of keeping a cat now on physical health of men and women of different age

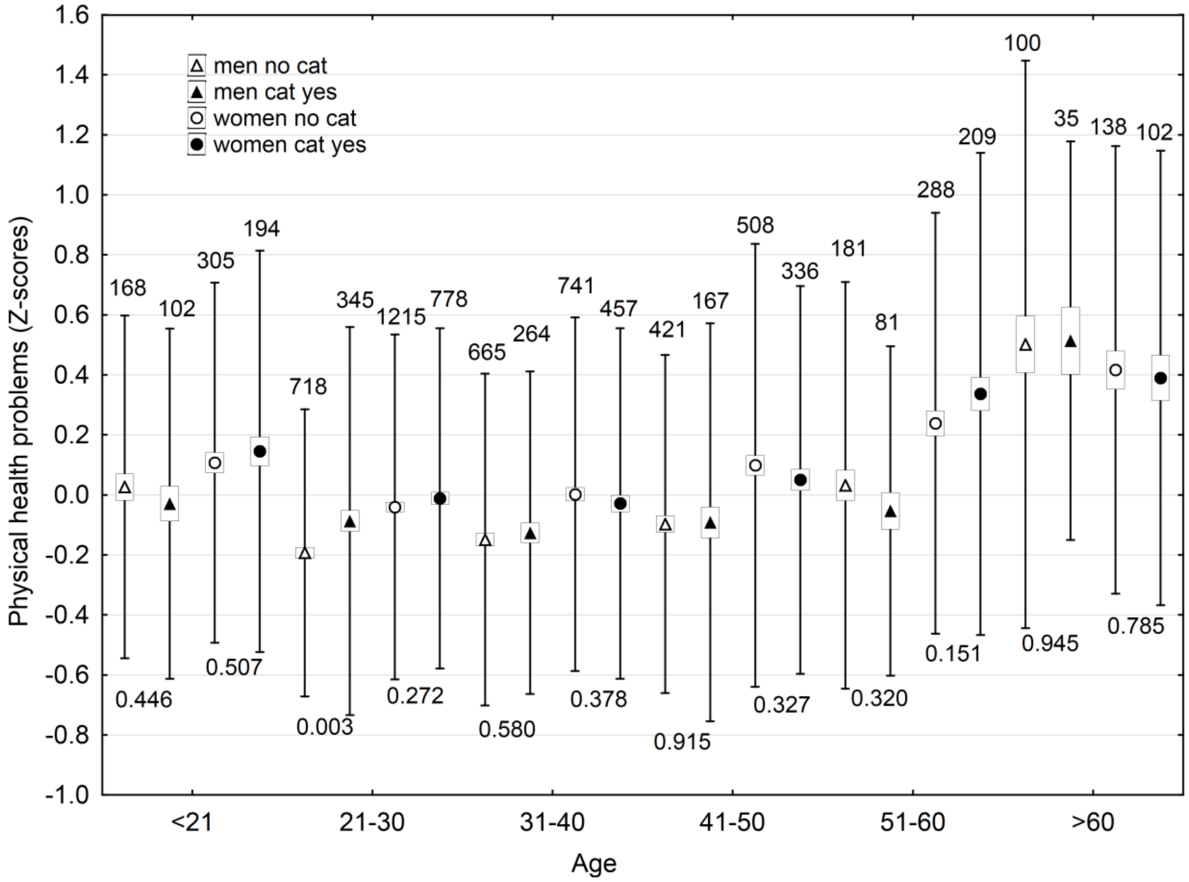

The boxes, spreads, upper numbers and lower numbers show standard errors, standard deviations, numbers of subjects in particular category and p-values of two-sided t-tests, respectively.
