# Supplementary material for: Friends with malefit. The effects of keeping dogs and cats, sustaining animal-related injuries and Toxoplasma infection on health and quality of life
Source: PLoS One. 2019 Nov 22;14(11):e0221988. doi: 10.1371/journal.pone.0221988 (PMC6874301; doi:10.1371/journal.pone.0221988)
Supplement: S15 Fig — The categories on x-axis describing the intensity of being injured by a pet are: 0- never, 1- only while playing, 2- only as a warning, 3- yes, minor injury (only skin cut), 4- yes, moderate injury (bleeding), 5- yes, serious injury, I had to seek medical treatment. (PDF) [file pone.0221988.s015.pdf]

Fig. 15. Association between intensity of sustained cat scratching and wellbeing – the scores of WHOQOL-BREF domains

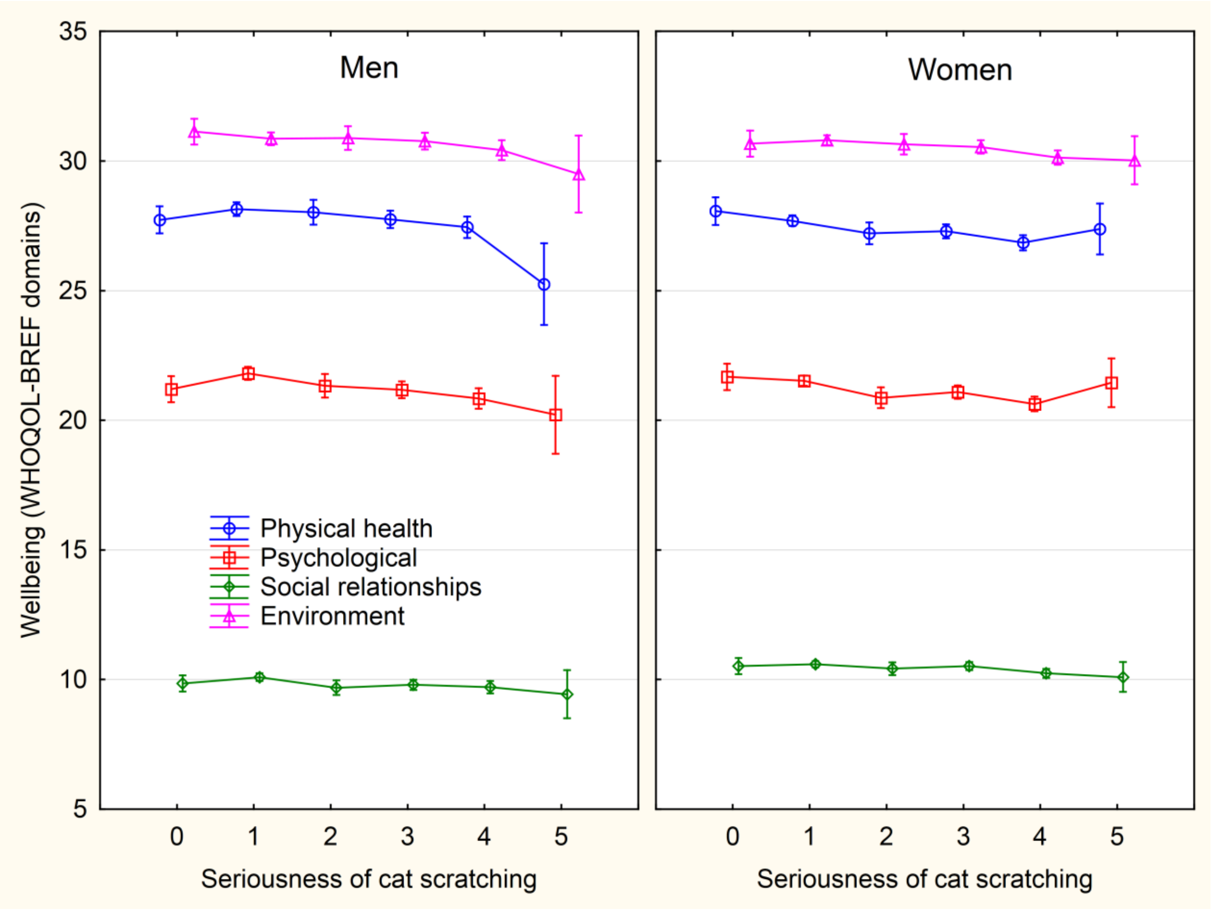

The categories on x-axis describing the intensity of being injured by a pet are: 0- never, 1- only while playing, 2- only as a warning, 3- yes, minor injury (only skin cut), 4- yes, moderate injury (bleeding), 5- yes, serious injury, I had to seek medical treatment.
