# Supplementary material for: Friends with malefit. The effects of keeping dogs and cats, sustaining animal-related injuries and Toxoplasma infection on health and quality of life
Source: PLoS One. 2019 Nov 22;14(11):e0221988. doi: 10.1371/journal.pone.0221988 (PMC6874301; doi:10.1371/journal.pone.0221988)
Supplement: S1 Table — The column 3 shows mean answer to particular questions and the columns 4–12 the percentage of subjects who provided particular answer (0–8). The questions on variables printed bold were responded by a code of the answer (for meaning of particular codes see the Material and methods), other questions by a number (e.g. 4 children). In such cases, the highest number of the scale (e.g. 8) always means "eight or more". The codes in parenthesis in the first column indicates whether particular variable correlate significantly with sex (s) age (a), education (e), and urbanization (u) in the whole population, men, and women, respectively. The code 0 means no correlation with sex, age education or urbanization. (PDF) [file pone.0221988.s016.pdf]

Table S1. Distribution of responses on particular questions of the questionnaire – categorical and ordinal variables

			Response to particular questions (%)								
		Mean	0	1	2	3	4	5	6	7	8
**education**	♂	5.31	1.03	4.56	5.15	29.31	11.89	12.43	30.90	4.73	
(All: s, a, u ♂: a, e, u ♀: a, e, u)	♀	5.41	1.02	4.50	3.62	26.22	13.61	14.12	33.17	3.74	
**urbanization**	♂	4.13	10.42	11.99	17.13	8.26	19.26	32.94			
(All: s, a, e ♂: a, e, u ♀: a, e, u)	♀	4.06	12.12	11.89	17.33	8.43	16.98	33.25			
**ever keeping a dog**	♂	0.69	30.82	69.18							
(All: s, a, e, u ♂: a, e, u ♀: a, e, u)	♀	0.75	24.75	75.25							
**ever keeping a cat**	♂	0.66	34.35	65.65							
(All: s, a, e, u ♂: a, e, u ♀: a, e, u)	♀	0.71	28.55	71.45							
**now keeping a dog**	♂	0.31	68.75	31.25							
(All: s, a, e, u ♂: a, e, u ♀: a, e, u)	♀	0.39	61.20	38.80							
**now keeping a cat**	♂	0.31	69.12	30.88							
(All: s, e, u ♂: a, e, u ♀: e, u)	♀	0.39	60.87	39.13							
number of dogs	♂	0.40	78.20	17.18	3.31	1.31					
(All: s, e ♂: 0 ♀: e)	♀	0.54	73.06	18.65	4.61	3.69					
number of cats	♂	0.53	56.02	26.11	9.65	8.23					
(All: s, a ♂: 0 ♀: a, u)	♀	0.72	49.80	28.35	8.81	13.03					
**biting by a dog**	♂	2.89	19.18	31.50	12.68	19.23	12.16	5.25			
(All: s ♂: a ♀: 0)	♀	2.72	24.81	32.00	10.49	16.55	10.89	5.25			
**biting by a cat**	♂	2.14	39.41	33.34	8.67	11.75	6.31	0.52			
(All: s, a ♂: 0 ♀: a)	♀	2.29	33.69	35.90	9.72	11.33	7.45	1.92			
**scratching by cat**	♂	3.02	9.72	37.93	11.40	23.78	15.98	1.18			
(All: s, a ♂: a ♀: a)	♀	3.13	6.26	40.65	9.48	22.63	19.33	1.64			
**toxoplasmosis**	♂	0.18	81.67	18.33							
(All: s, a, u ♂: 0 ♀: a, u)	♀	0.27	72.91	27.09							
**smoking,**	♂	2.14	70.53	3.89	2.48	3.71	6.96	8.62	3.50	0.31	
(All: s, a, e, u ♂: a ♀: 0)	♀	1.90	75.07	4.09	2.18	3.32	7.44	6.08	1.76	0.06	
**consuming alcohol**	♂	3.45	17.35	17.96	12.70	21.85	18.30	8.48	3.34	0.03	
(All: s, a, e, u ♂: e, u ♀: a, e, u)	♀	2.92	25.70	22.54	13.63	18.83	13.00	4.58	1.70	0.02	
**consumming illegal drugs**	♂	1.50	80.93	8.19	2.51	2.67	1.90	1.50	2.02	0.28	
(All: s, a, e, u ♂: a, e, u ♀: a, e, u)	♀	1.22	89.52	5.58	1.58	1.33	0.87	0.46	0.65	0.02	
**physical health problems**	♂	3.70	5.88	12.26	25.95	28.83	15.83	11.24			
(All: s, a, e ♂: a, e ♀: a, e)	♀	3.91	4.00	11.18	23.48	27.09	19.72	14.52			
**mental health problems**	♂	3.23	7.54	21.82	33.19	21.49	9.12	6.84			
(All: s, a, e ♂: a, e ♀: a, e)	♀	3.51	4.85	16.71	32.39	24.23	12.27	9.54			
**family situation**	♂	4.57	3.12	4.82	11.46	21.00	31.67	27.93			
(All: s, e ♂: a, e, u ♀: e)	♀	4.74	1.93	4.60	9.45	19.28	31.12	33.62			
**economic situation**	♂	4.32	2.70	5.02	12.21	33.04	31.47	15.56			
(All: s, a, e, u ♂: a, e, u ♀: a, e, u)	♀	4.74	1.86	4.40	13.73	36.40	29.96	13.66			
drugs prescribed	♂	1.62	70.59	14.47	6.62	3.83	2.36	0.74	0.40	0.34	0.64
(All: s, a, u ♂: a, e, u ♀: a, u)	♀	1.64	65.91	19.45	7.31	3.97	1.46	0.78	0.38	0.23	0.51
