# Supplementary material for: Friends with malefit. The effects of keeping dogs and cats, sustaining animal-related injuries and Toxoplasma infection on health and quality of life
Source: PLoS One. 2019 Nov 22;14(11):e0221988. doi: 10.1371/journal.pone.0221988 (PMC6874301; doi:10.1371/journal.pone.0221988)
Supplement: S2 Table — For the explanation of codes in the column 1 see the Table 2. (PDF) [file pone.0221988.s017.pdf]

Table S2 Continuous depending variables - difference between men and women.

	Mean Men	Mean Women	t-value	d.f.	p
Wellbeing (WHOQOL-BREF)<br>(All: a, e ♂: a ♀: a, e)	97.267	97.091	0.536	7482	0.592
Mental health problems score<br>(All: s, a, u ♂: a, e ♀: a, e)	-0.082	0.049	-10.346	8583	0.000
Physical health problems score<br>(All: s, a, e, u ♂: a ♀: a, e)	-0.089	0.057	-10.469	8539	0.000
Body Mass Index<br>(All: s, a, u ♂: a, e ♀: a)	26.061	24.519	15.037	10771	0.000
Intensity of liking dogs<br>(All: s, a, e, u ♂: e, u ♀: a, e, u)	67.858	76.669	-14.516	9482	0.000
Intensity of liking cats<br>(All: s, a, e ♂: a, e ♀: e)	61.225	71.854	-15.926	9453	0.000
Preferrence dogs to cats<br>(All: s, e, u ♂: u ♀: u)	6.646	4.928	2.053	9391	0.040
Sexual activity<br>(All: s, a, u ♂: a, u ♀: a, u)	-0.024	0.026	-2.869	7373	0.004
Sexual desire<br>(All: s, e ♂: a, e, u ♀: 0)	7.266	6.975	6.806	7242	0.000

For the explanation of codes in the column 1 see the table 2.
