# Supplementary material for: Friends with malefit. The effects of keeping dogs and cats, sustaining animal-related injuries and Toxoplasma infection on health and quality of life
Source: PLoS One. 2019 Nov 22;14(11):e0221988. doi: 10.1371/journal.pone.0221988 (PMC6874301; doi:10.1371/journal.pone.0221988)
Supplement: S3 Table — (PDF) [file pone.0221988.s018.pdf]

Table S3: Partial Kendall correlation (age, education, and urbanization controlled) between variables listed in the first raw and first column.

MEN

a) Partial Kendall Tau (significant Tau printed bold, no correction for multiple comparission. Blue cells and red cells indicate negative and positive correlation, respectively.)

	like dogs	like cats	refer dog	dog ever	dog now	ogs numb	dog bit	cat ever	cat now	ats numb	cat bit	at scratche	smoking	alcohol	egal dru	BMI
WHOQOL-BREF health	**0.050**	0.013	0.020	0.002	0.015	-0.019	-0.017	-0.002	0.006	0.024	**-0.035**	**-0.037**	**-0.040**	0.013	**-0.029**	**-0.081**
WHOQOL-BREF psychological	**0.080**	0.012	**0.038**	**0.033**	**0.034**	-0.006	-0.001	-0.011	0.005	0.009	**-0.032**	**-0.044**	**-0.039**	-0.003	**-0.059**	**-0.034**
WHOQOL-BREF social relationships	**0.062**	0.008	**0.027**	0.021	**0.038**	**0.064**	0.022	0.004	0.020	-0.033	**-0.029**	**-0.034**	0.001	0.014	-0.016	**-0.037**
WHOQOL-BREF environment	0.008	**0.037**	**-0.031**	-0.020	-0.003	-0.026	**-0.026**	-0.014	0.011	-0.018	-0.021	**-0.043**	**-0.045**	**0.037**	-0.011	**-0.050**
WHOQOL-BREF total score	**0.063**	0.021	0.018	0.009	0.019	-0.006	-0.011	-0.009	0.009	-0.012	**-0.036**	**-0.048**	**-0.046**	0.022	**-0.035**	**-0.067**
children	**-0.054**	**-0.082**	**0.032**	0.021	0.021	0.032	-0.015	0.016	0.000	-0.002	**-0.030**	**-0.022**	-0.001	-0.001	**-0.076**	**0.099**
siblings	**-0.032**	**-0.024**	-0.011	**0.047**	0.003	**0.040**	-0.001	**0.050**	**0.027**	0.021	0.018	0.013	-0.017	-0.018	**-0.023**	-0.012
family situation	**0.028**	-0.006	0.021	-0.007	**0.036**	0.034	-0.010	-0.003	0.000	-0.025	**-0.047**	**-0.065**	-0.022	**0.033**	**-0.037**	0.001
economic situation	-0.002	-0.016	0.010	**-0.036**	-0.008	-0.023	**-0.026**	**-0.037**	**-0.023**	-0.020	**-0.025**	**-0.027**	**-0.105**	0.013	**-0.077**	0.010
drugs prescribed	-0.011	-0.004	-0.009	-0.009	0.005	**0.104**	**-0.025**	**-0.043**	-0.001	0.040	-0.013	0.000	**-0.024**	**-0.057**	**-0.073**	**0.127**
drugs non-prescribed	**0.028**	0.010	0.015	**0.042**	0.010	0.006	0.009	0.000	-0.005	0.008	**0.029**	**0.034**	-0.021	-0.003	**0.050**	-0.012
practical doctor visits	0.003	-0.008	0.009	-0.013	-0.003	**0.073**	-0.005	-0.019	0.021	-0.013	0.003	0.014	**-0.051**	**-0.050**	**-0.040**	**0.054**
antibiotics	-0.022	-0.021	0.000	-0.022	0.007	**0.073**	0.001	-0.010	**0.024**	0.016	0.003	**0.029**	-0.023	**-0.031**	-0.012	**0.032**
medical specialists visited	-0.014	-0.006	-0.004	-0.017	**0.027**	0.025	0.022	0.009	0.018	-0.028	**0.052**	**0.062**	**-0.038**	**-0.044**	-0.022	**0.027**
anxiety	**-0.032**	**0.035**	**-0.048**	-0.016	0.003	-0.001	**0.024**	0.007	**0.032**	**-0.045**	**0.055**	**0.057**	**0.053**	0.016	**0.089**	-0.023
phobia	**-0.050**	**0.038**	**-0.064**	-0.024	-0.004	0.015	0.014	-0.001	**0.040**	0.007	**0.047**	**0.033**	0.005	0.019	**0.050**	-0.001
depression	-0.024	**0.035**	**-0.041**	0.017	0.023	0.019	**0.042**	**0.026**	**0.030**	-0.001	**0.082**	**0.061**	**0.074**	**0.039**	**0.107**	0.013
mania	**-0.028**	**0.033**	**-0.040**	0.010	0.014	0.031	**0.066**	**0.039**	**0.052**	-0.020	**0.086**	**0.051**	**0.068**	**0.062**	**0.150**	**-0.036**
obsession	**-0.060**	**0.028**	**-0.058**	-0.024	-0.016	-0.009	0.021	**0.031**	0.014	0.042	**0.040**	**0.061**	0.013	**0.040**	**0.071**	**-0.028**
audial hallucination	**-0.028**	-0.005	-0.010	-0.002	0.004	-0.001	0.021	0.014	0.017	-0.044	**0.042**	**0.037**	**0.032**	**0.033**	**0.096**	-0.022
visual halucination	**-0.034**	-0.007	-0.014	-0.009	0.006	0.005	0.013	0.001	0.018	0.040	**0.039**	**0.028**	0.022	**0.040**	**0.080**	**-0.029**
headache	-0.002	0.014	-0.019	-0.005	0.012	0.010	**0.030**	0.016	0.015	0.017	**0.032**	**0.041**	-0.018	**-0.029**	-0.004	**0.030**
subjective physical health problems	**-0.035**	0.017	**-0.042**	**-0.026**	-0.009	0.017	**-0.036**	-0.019	-0.013	0.000	0.004	0.019	**0.083**	**-0.028**	**-0.031**	**0.176**
subjective mental health problems	**-0.044**	0.013	**-0.042**	**-0.035**	-0.012	0.001	-0.006	-0.014	-0.014	-0.021	0.008	0.015	**0.047**	-0.001	0.014	0.018
diagnosed psychiatric disorders	0.000	**0.042**	**-0.036**	**0.028**	0.007	0.024	**0.049**	**0.030**	**0.028**	0.030	**0.074**	**0.054**	**0.086**	**-0.048**	**0.044**	**0.032**
non-diagnosed psychiatric disorders	-0.016	**0.036**	**-0.037**	0.006	-0.007	-0.021	**0.042**	0.004	0.009	0.008	**0.065**	**0.050**	**0.090**	**0.039**	**0.100**	0.010
psychiatric disorders total number	-0.012	**0.048**	**-0.047**	0.021	-0.002	-0.002	**0.056**	0.018	**0.028**	0.014	**0.091**	**0.073**	**0.110**	0.006	**0.099**	**0.024**
partner's diagnosed psychiatric disorders	0.000	0.008	0.000	**0.036**	0.022	0.034	**0.038**	0.016	**0.036**	0.008	**0.047**	**0.067**	**0.025**	**0.023**	**0.057**	0.013
partner's non-diagnosed psychiatric disord.	-0.002	0.009	-0.002	0.007	**0.033**	**0.049**	**0.031**	**0.034**	**0.055**	**0.124**	**0.027**	**0.025**	**0.037**	-0.005	0.021	0.000
partner's psychiatric disord. total number	-0.005	0.008	-0.004	**0.026**	**0.026**	0.027	**0.050**	**0.032**	**0.060**	**0.069**	**0.040**	**0.061**	**0.032**	0.019	**0.048**	0.010
mental health problems score	**-0.025**	**0.044**	**-0.056**	-0.004	0.006	0.009	**0.044**	0.018	**0.044**	0.005	**0.089**	**0.076**	**0.067**	0.009	**0.103**	-0.003
physical health problems score	0.008	-0.004	0.008	0.001	0.012	**0.079**	0.000	-0.013	0.018	-0.006	0.023	**0.042**	**-0.039**	**-0.056**	-0.020	**0.059**
sexual activity	**0.083**	0.003	**0.049**	**0.102**	0.013	**0.045**	**0.085**	**0.062**	0.012	0.008	**0.072**	**0.052**	**0.222**	**0.114**	**0.157**	**0.046**
sexual desire	**0.075**	0.017	**0.043**	0.012	**0.036**	0.002	0.004	-0.006	-0.015	0.000	-0.009	0.001	-0.001	**0.043**	-0.016	**0.028**

b) p-values of two-sided tests

	like dogs	like cats	refer dog	dog ever	dog now	ogs numb	dog bit	cat ever	cat now	ats numb	cat bit	at scratche	smoking	alcohol	egal dru	BMI
WHOQOL-BREF health	0.000	0.288	0.098	0.851	0.225	0.385	0.172	0.849	0.629	0.267	0.005	0.002	0.001	0.287	0.019	0.000
WHOQOL-BREF psychological	0.000	0.333	0.002	0.007	0.006	0.804	0.947	0.385	0.665	0.678	0.010	0.000	0.002	0.825	0.000	0.006
WHOQOL-BREF social relationships	0.000	0.491	0.026	0.083	0.002	0.004	0.076	0.760	0.099	0.137	0.020	0.006	0.921	0.266	0.198	0.002
WHOQOL-BREF environment	0.490	0.003	0.013	0.109	0.787	0.249	0.031	0.238	0.377	0.416	0.087	0.000	0.000	0.002	0.379	0.000
WHOQOL-BREF total score	0.000	0.087	0.164	0.477	0.135	0.776	0.387	0.471	0.482	0.591	0.004	0.000	0.000	0.080	0.006	0.000
children	0.000	0.000	0.004	0.061	0.058	0.105	0.176	0.146	0.984	0.922	0.007	0.046	0.910	0.954	0.000	0.000
siblings	0.003	0.030	0.311	0.000	0.818	0.043	0.898	0.000	0.016	0.298	0.101	0.251	0.151	0.134	0.044	0.260
family situation	0.013	0.579	0.058	0.549	0.001	0.081	0.345	0.818	0.967	0.212	0.000	0.000	0.059	0.004	0.002	0.931
economic situation	0.851	0.153	0.392	0.001	0.468	0.240	0.018	0.001	0.036	0.314	0.026	0.013	0.000	0.268	0.000	0.308
drugs prescribed	0.362	0.728	0.429	0.464	0.653	0.000	0.031	0.000	0.930	0.060	0.256	0.979	0.041	0.000	0.000	0.000
drugs non-prescribed	0.020	0.416	0.212	0.000	0.394	0.768	0.434	0.981	0.691	0.707	0.014	0.004	0.079	0.767	0.000	0.307
practical doctor visits	0.812	0.471	0.452	0.261	0.777	0.001	0.642	0.106	0.079	0.549	0.816	0.237	0.000	0.000	0.001	0.000
antibiotics	0.063	0.081	0.974	0.055	0.524	0.001	0.936	0.389	0.044	0.463	0.817	0.015	0.052	0.008	0.318	0.007
medical specialists visited	0.248	0.633	0.761	0.154	0.021	0.248	0.065	0.459	0.133	0.182	0.000	0.000	0.001	0.000	0.058	0.021
anxiety	0.008	0.004	0.000	0.199	0.786	0.949	0.049	0.567	0.009	0.042	0.000	0.000	0.000	0.182	0.000	0.055
phobia	0.000	0.003	0.000	0.057	0.719	0.507	0.247	0.945	0.001	0.765	0.000	0.009	0.664	0.134	0.000	0.906
depression	0.053	0.005	0.001	0.168	0.057	0.408	0.001	0.036	0.014	0.961	0.000	0.000	0.000	0.001	0.000	0.278
mania	0.029	0.011	0.002	0.428	0.274	0.190	0.000	0.002	0.000	0.398	0.000	0.000	0.000	0.000	0.000	0.005
obsession	0.000	0.027	0.000	0.058	0.204	0.701	0.105	0.014	0.263	0.071	0.002	0.000	0.294	0.001	0.000	0.029
audial hallucination	0.037	0.695	0.447	0.875	0.770	0.964	0.118	0.291	0.205	0.071	0.001	0.005	0.014	0.012	0.000	0.100
visual halucination	0.010	0.612	0.281	0.498	0.665	0.850	0.313	0.960	0.180	0.098	0.003	0.035	0.103	0.003	0.000	0.027
headache	0.902	0.263	0.120	0.667	0.325	0.646	0.013	0.206	0.223	0.440	0.010	0.001	0.146	0.020	0.733	0.015
subjective physical health problems	0.004	0.157	0.001	0.030	0.449	0.448	0.003	0.113	0.291	0.994	0.761	0.123	0.000	0.019	0.010	0.000
subjective mental health problems	0.000	0.275	0.001	0.004	0.342	0.949	0.593	0.256	0.241	0.334	0.488	0.204	0.000	0.928	0.263	0.143
diagnosed psychiatric disorders	0.985	0.000	0.003	0.016	0.523	0.263	0.000	0.009	0.015	0.159	0.000	0.000	0.000	0.000	0.000	0.006
non-diagnosed psychiatric disorders	0.166	0.002	0.002	0.628	0.561	0.311	0.000	0.727	0.447	0.716	0.000	0.000	0.000	0.001	0.000	0.384
psychiatric disorders total number	0.319	0.000	0.000	0.076	0.880	0.936	0.000	0.130	0.019	0.526	0.000	0.000	0.000	0.584	0.000	0.039
partner's diagnosed psychiatric disorders	0.999	0.509	0.968	0.002	0.059	0.112	0.001	0.160	0.002	0.716	0.000	0.000	0.031	0.046	0.000	0.276
partner's non-diagnosed psychiatric disord.	0.843	0.424	0.853	0.538	0.004	0.021	0.007	0.003	0.000	0.000	0.020	0.030	0.002	0.665	0.069	0.994
partner's psychiatric disord. total number	0.652	0.511	0.721	0.028	0.027	0.196	0.000	0.006	0.000	0.001	0.001	0.000	0.007	0.102	0.000	0.410
mental health problems score	0.029	0.000	0.000	0.762	0.595	0.684	0.000	0.122	0.000	0.822	0.000	0.000	0.000	0.418	0.000	0.826
physical health problems score	0.492	0.749	0.496	0.946	0.320	0.000	0.987	0.266	0.135	0.785	0.054	0.000	0.001	0.000	0.094	0.000
sexual activity	0.000	0.784	0.000	0.000	0.299	0.045	0.000	0.000	0.337	0.733	0.000	0.000	0.000	0.000	0.000	0.000
sexual desire	0.000	0.174	0.001	0.326	0.003	0.915	0.751	0.607	0.231	0.999	0.465	0.926	0.952	0.001	0.201	0.023
