# Supplementary material for: Friends with malefit. The effects of keeping dogs and cats, sustaining animal-related injuries and Toxoplasma infection on health and quality of life
Source: PLoS One. 2019 Nov 22;14(11):e0221988. doi: 10.1371/journal.pone.0221988 (PMC6874301; doi:10.1371/journal.pone.0221988)
Supplement: S4 Table — (PDF) [file pone.0221988.s019.pdf]

Table S4: Partial Kendall correlation (age, education, and urbanization controlled) between variables listed in the first raw and first column.

WOMEN

a) Partial Kendall Tau (significant Tau printed bold, no correction for multiple comparission. Blue cells and red cells indicate negative and positive correlation, respectively.)

	like dogs	like cats	refer dog	dog ever	dog now	ogs numb	dog bit	cat ever	cat now	ats numb	cat bit	scratch	smoking	alcohol	legal dru	BMI
WHOQOL-BREF health	**0.032**	0.005	**0.021**	-0.010	0.003	**-0.046**	**-0.029**	**-0.038**	**-0.020**	**-0.035**	**-0.036**	**-0.065**	-0.008	**0.028**	-0.010	**-0.068**
WHOQOL-BREF psychological	0.008	0.002	0.003	-0.002	-0.001	-0.023	**-0.027**	-0.014	-0.005	-0.024	**-0.039**	**-0.051**	**-0.045**	**-0.032**	**-0.042**	**-0.054**
WHOQOL-BREF social relationships	0.017	0.018	-0.006	-0.005	-0.004	-0.028	**-0.030**	0.001	-0.004	-0.021	**-0.031**	**-0.034**	**-0.019**	0.006	-0.008	**-0.041**
WHOQOL-BREF environment	0.002	0.014	-0.008	**-0.040**	**-0.024**	**-0.060**	**-0.052**	**-0.036**	-0.015	**-0.049**	**-0.037**	**-0.047**	**-0.025**	**0.032**	**-0.029**	**-0.030**
WHOQOL-BREF total score	0.019	0.007	0.009	-0.019	-0.006	**-0.046**	**-0.042**	**-0.034**	-0.016	**-0.044**	**-0.044**	**-0.063**	**-0.034**	0.014	**-0.032**	**-0.064**
children	**-0.095**	**-0.080**	-0.001	**0.020**	**-0.026**	-0.027	-0.009	**0.029**	**-0.023**	**-0.040**	**-0.056**	**-0.057**	**-0.046**	**-0.077**	**-0.058**	**0.066**
siblings	**-0.054**	**-0.048**	0.003	0.009	-0.014	0.004	-0.016	**0.036**	0.011	-0.008	**-0.028**	**-0.025**	**-0.032**	**-0.034**	**-0.025**	0.005
family situation	0.017	-0.001	0.008	**-0.036**	-0.009	-0.013	**-0.033**	**-0.018**	-0.014	-0.009	**-0.028**	**-0.034**	**-0.066**	-0.016	**-0.035**	**-0.031**
economic situation	**-0.023**	**-0.032**	0.013	**-0.045**	**-0.029**	**-0.034**	**-0.046**	**-0.060**	**-0.041**	**-0.057**	**-0.043**	**-0.050**	**-0.079**	-0.018	**-0.049**	**-0.042**
drugs prescribed	0.002	0.000	0.004	0.002	0.003	0.022	-0.004	0.001	0.003	**0.030**	-0.008	0.014	**-0.030**	**-0.071**	**-0.036**	**0.113**
drugs non-prescribed	**0.029**	0.008	0.013	0.018	0.007	0.005	**0.032**	0.012	0.011	-0.012	-0.001	0.001	**-0.030**	-0.015	**0.034**	-0.013
practical doctor visits	0.007	0.008	-0.005	-0.003	-0.018	0.017	**0.021**	0.013	-0.004	0.009	**0.019**	**0.036**	**-0.019**	-0.017	0.008	**0.063**
antibiotics	0.009	0.001	0.002	-0.001	0.003	**0.030**	**0.025**	-0.007	0.011	0.014	**0.024**	**0.038**	**0.033**	**0.026**	**0.029**	**0.042**
medical specialists visited	0.013	-0.001	0.010	-0.002	-0.002	-0.017	**0.026**	-0.007	0.011	0.023	**0.029**	**0.047**	-0.010	-0.012	0.006	**0.046**
anxiety	-0.009	0.018	-0.014	0.008	-0.006	0.006	**0.046**	0.013	**0.024**	0.008	**0.050**	**0.073**	**0.056**	**0.052**	**0.049**	0.003
phobia	-0.017	**0.024**	**-0.027**	0.007	0.010	0.000	**0.041**	0.013	**0.038**	0.008	**0.024**	**0.046**	**0.034**	**0.026**	0.010	**0.026**
depression	-0.006	**0.039**	**-0.032**	**0.027**	0.001	0.030	**0.060**	**0.044**	**0.046**	0.020	**0.065**	**0.071**	**0.075**	**0.074**	**0.080**	**0.036**
mania	**-0.051**	-0.010	**-0.026**	**0.042**	0.001	-0.005	**0.057**	**0.034**	**0.028**	0.021	**0.051**	**0.067**	**0.093**	**0.080**	**0.114**	**0.026**
obsession	**-0.046**	-0.008	**-0.020**	**0.025**	-0.003	-0.017	**0.055**	-0.001	**0.026**	0.001	**0.043**	**0.067**	**0.045**	**0.052**	**0.066**	0.005
audial hallucination	**-0.036**	-0.008	-0.011	**0.039**	0.004	0.031	**0.052**	**0.027**	**0.022**	**0.035**	**0.035**	**0.055**	**0.062**	**0.034**	**0.083**	0.011
visual halucination	**-0.037**	-0.014	-0.010	**0.035**	-0.002	0.023	**0.057**	**0.029**	**0.026**	**0.046**	**0.032**	**0.056**	**0.060**	0.015	**0.072**	**0.024**
headache	-0.002	**0.031**	**-0.022**	**0.023**	0.009	0.022	**0.034**	**0.022**	**0.042**	0.016	**0.041**	**0.060**	**0.031**	0.016	**0.025**	0.016
subjective physical health problems	-0.013	0.010	**-0.025**	-0.012	-0.002	0.019	**-0.019**	0.008	0.018	0.029	0.007	**0.038**	**0.034**	**-0.045**	-0.004	**0.222**
subjective mental health problems	-0.005	0.004	-0.008	-0.002	-0.016	0.012	0.004	-0.001	-0.010	0.016	0.018	**0.034**	**0.030**	0.009	0.014	**0.026**
diagnosed psychiatric disorders	0.010	**0.048**	**-0.035**	**0.042**	**0.018**	0.009	**0.061**	**0.053**	**0.053**	0.020	**0.080**	**0.079**	**0.090**	**-0.023**	**0.030**	**0.058**
non-diagnosed psychiatric disorders	-0.006	**0.045**	**-0.039**	0.015	0.004	0.028	**0.047**	**0.049**	**0.049**	0.024	**0.064**	**0.050**	**0.079**	**0.045**	**0.071**	**0.031**
psychiatric disorders total number	0.000	**0.055**	**-0.046**	**0.030**	0.013	0.014	**0.070**	**0.055**	**0.060**	**0.030**	**0.085**	**0.074**	**0.105**	**0.024**	**0.067**	**0.051**
partner's diagnosed psychiatric disorders	-0.004	0.002	-0.004	**0.028**	0.013	-0.016	0.008	**0.025**	0.003	0.028	0.008	-0.011	**0.030**	0.008	**0.047**	**0.027**
partner's non-diagnosed psychiatric disord.	-0.003	**0.024**	**-0.019**	0.014	0.002	-0.005	-0.017	**0.046**	0.014	0.021	0.017	0.005	**0.020**	-0.003	0.011	**0.047**
partner's psychiatric disord. total number	0.000	**0.019**	-0.013	**0.025**	0.012	-0.010	0.003	**0.041**	0.013	**0.031**	0.014	-0.004	**0.029**	0.010	**0.044**	**0.034**
mental health problems score	**-0.019**	**0.044**	**-0.043**	**0.022**	0.005	0.019	**0.072**	**0.040**	**0.053**	**0.033**	**0.083**	**0.100**	**0.084**	**0.052**	**0.075**	**0.032**
physical health problems score	**0.021**	0.013	0.004	0.008	-0.004	0.004	**0.030**	0.010	0.009	0.018	**0.022**	**0.040**	-0.011	**-0.023**	0.010	**0.072**
sexual activity	**0.038**	**0.041**	-0.003	**0.072**	0.016	**0.038**	**0.052**	**0.047**	**0.029**	0.026	**0.056**	**0.042**	**0.248**	**0.150**	**0.158**	**0.021**
sexual desire	**0.103**	**0.033**	**0.045**	-0.013	0.013	**0.058**	0.015	**-0.026**	**-0.025**	-0.031	**-0.024**	**-0.041**	**0.028**	**0.023**	0.006	-0.001

b) p-values of two-sided tests

	like dogs	like cats	refer dog	dog ever	dog now	ogs numb	dog bit	cat ever	cat now	ats numb	cat bit	scratch	smoking	alcohol	legal dru	BMI
WHOQOL-BREF health	0.001	0.596	0.031	0.318	0.739	0.004	0.003	0.000	0.039	0.024	0.000	0.000	0.423	0.004	0.285	0.000
WHOQOL-BREF psychological	0.399	0.820	0.725	0.821	0.950	0.152	0.004	0.158	0.595	0.115	0.000	0.000	0.000	0.001	0.000	0.000
WHOQOL-BREF social relationships	0.083	0.059	0.533	0.594	0.650	0.080	0.002	0.911	0.666	0.176	0.001	0.001	0.047	0.551	0.392	0.000
WHOQOL-BREF environment	0.855	0.148	0.422	0.000	0.013	0.000	0.000	0.000	0.112	0.001	0.000	0.000	0.010	0.001	0.003	0.002
WHOQOL-BREF total score	0.053	0.485	0.387	0.055	0.538	0.004	0.000	0.001	0.109	0.005	0.000	0.000	0.000	0.142	0.001	0.000
children	0.000	0.000	0.916	0.021	0.002	0.052	0.294	0.001	0.008	0.004	0.000	0.000	0.000	0.000	0.000	0.000
siblings	0.000	0.000	0.704	0.298	0.103	0.758	0.065	0.000	0.192	0.565	0.001	0.004	0.001	0.000	0.007	0.540
family situation	0.055	0.948	0.351	0.000	0.278	0.371	0.000	0.043	0.107	0.514	0.001	0.000	0.000	0.085	0.000	0.000
economic situation	0.008	0.000	0.141	0.000	0.001	0.015	0.000	0.000	0.000	0.000	0.000	0.000	0.000	0.053	0.000	0.000
drugs prescribed	0.850	0.971	0.637	0.789	0.709	0.137	0.671	0.908	0.706	0.041	0.377	0.141	0.001	0.000	0.000	0.000
drugs non-prescribed	0.002	0.408	0.164	0.051	0.431	0.752	0.001	0.179	0.224	0.400	0.922	0.889	0.001	0.101	0.000	0.149
practical doctor visits	0.436	0.385	0.594	0.780	0.056	0.248	0.025	0.153	0.697	0.541	0.043	0.000	0.041	0.062	0.412	0.000
antibiotics	0.351	0.925	0.792	0.898	0.705	0.042	0.007	0.419	0.225	0.324	0.011	0.000	0.000	0.005	0.002	0.000
medical specialists visited	0.166	0.878	0.283	0.841	0.840	0.268	0.005	0.423	0.243	0.111	0.002	0.000	0.267	0.189	0.536	0.000
anxiety	0.320	0.064	0.152	0.410	0.518	0.690	0.000	0.163	0.010	0.593	0.000	0.000	0.000	0.000	0.000	0.716
phobia	0.085	0.014	0.005	0.445	0.280	0.994	0.000	0.168	0.000	0.591	0.012	0.000	0.000	0.006	0.286	0.007
depression	0.507	0.000	0.001	0.004	0.947	0.056	0.000	0.000	0.000	0.184	0.000	0.000	0.000	0.000	0.000	0.000
mania	0.000	0.338	0.010	0.000	0.884	0.763	0.000	0.001	0.005	0.190	0.000	0.000	0.000	0.000	0.000	0.009
obsession	0.000	0.413	0.041	0.010	0.800	0.283	0.000	0.883	0.007	0.968	0.000	0.000	0.000	0.000	0.000	0.644
audial hallucination	0.000	0.438	0.286	0.000	0.693	0.056	0.000	0.008	0.028	0.029	0.000	0.000	0.000	0.001	0.000	0.268
visual halucination	0.000	0.174	0.332	0.001	0.846	0.172	0.000	0.004	0.009	0.005	0.002	0.000	0.000	0.147	0.000	0.017
headache	0.850	0.001	0.021	0.016	0.355	0.159	0.000	0.019	0.000	0.303	0.000	0.000	0.001	0.090	0.008	0.091
subjective physical health problems	0.163	0.279	0.011	0.209	0.851	0.233	0.049	0.401	0.065	0.059	0.453	0.000	0.000	0.000	0.684	0.000
subjective mental health problems	0.588	0.702	0.427	0.831	0.090	0.425	0.702	0.911	0.280	0.293	0.058	0.000	0.002	0.330	0.160	0.008
diagnosed psychiatric disorders	0.296	0.000	0.000	0.000	0.046	0.559	0.000	0.000	0.000	0.171	0.000	0.000	0.000	0.013	0.001	0.000
non-diagnosed psychiatric disorders	0.509	0.000	0.000	0.098	0.673	0.063	0.000	0.000	0.000	0.100	0.000	0.000	0.000	0.000	0.000	0.001
psychiatric disorders total number	0.963	0.000	0.000	0.001	0.159	0.356	0.000	0.000	0.000	0.042	0.000	0.000	0.000	0.010	0.000	0.000
partner's diagnosed psychiatric disorders	0.628	0.837	0.697	0.003	0.173	0.274	0.372	0.007	0.721	0.056	0.383	0.238	0.001	0.385	0.000	0.004
partner's non-diagnosed psychiatric disord.	0.733	0.008	0.039	0.128	0.826	0.743	0.067	0.000	0.127	0.148	0.062	0.604	0.027	0.718	0.240	0.000
partner's psychiatric disord. total number	0.966	0.036	0.160	0.006	0.189	0.508	0.760	0.000	0.149	0.038	0.128	0.629	0.002	0.271	0.000	0.000
mental health problems score	0.042	0.000	0.000	0.016	0.571	0.197	0.000	0.000	0.000	0.024	0.000	0.000	0.000	0.000	0.000	0.000
physical health problems score	0.023	0.148	0.647	0.395	0.691	0.787	0.001	0.271	0.349	0.213	0.015	0.000	0.248	0.012	0.271	0.000
sexual activity	0.000	0.000	0.752	0.000	0.118	0.019	0.000	0.000	0.004	0.105	0.000	0.000	0.000	0.000	0.000	0.037
sexual desire	0.000	0.001	0.000	0.214	0.207	0.001	0.135	0.012	0.013	0.055	0.020	0.000	0.006	0.023	0.574	0.959
