# Supplementary material for: Friends with malefit. The effects of keeping dogs and cats, sustaining animal-related injuries and Toxoplasma infection on health and quality of life
Source: PLoS One. 2019 Nov 22;14(11):e0221988. doi: 10.1371/journal.pone.0221988 (PMC6874301; doi:10.1371/journal.pone.0221988)
Supplement: S5 Table — (PDF) [file pone.0221988.s020.pdf]

Table S5: Partial Kendall correlation (age, education, and urbanization controlled) between variables listed in the first raw and first column.

MEN WHO NEVER KEPT A DOG

a) Partial Kendall Tau (significant Tau printed bold, no correction for multiple comparission. Blue cells and red cells indicate negative and positive correlation, respectively.)

	like dogs	like cats	refer dog	dog bit	cat ever	cat now	ats numbe	cat bit	scratch	smoking	alcohol	egal dru	BMI
WHOQOL-BREF health	0.020	0.035	-0.019	0.002	-0.023	0.023	0.036	-0.030	**-0.046**	**-0.055**	0.009	0.012	**-0.048**
WHOQOL-BREF psychological	0.035	-0.002	0.020	0.033	-0.039	0.014	0.059	-0.001	-0.022	**-0.044**	0.002	0.017	-0.019
WHOQOL-BREF social relationships	0.029	0.005	0.018	0.024	-0.040	0.002	-0.037	-0.011	-0.022	0.000	0.041	0.034	-0.016
WHOQOL-BREF environment	**-0.049**	0.041	**-0.068**	-0.031	-0.017	**0.062**	-0.060	0.021	-0.004	-0.033	0.032	0.005	**-0.051**
WHOQOL-BREF total score	0.011	0.028	-0.025	0.001	-0.037	0.031	-0.022	-0.008	-0.040	**-0.048**	0.031	0.024	**-0.049**
children	**-0.060**	**-0.074**	0.011	0.008	0.008	-0.015	-0.003	0.002	0.009	**0.070**	0.003	-0.025	**0.094**
siblings	**-0.061**	**-0.065**	-0.003	-0.037	-0.011	-0.017	0.000	-0.024	0.008	-0.011	0.002	-0.008	-0.009
family situation	0.020	0.011	0.002	**0.044**	0.011	0.001	-0.072	-0.021	**-0.059**	-0.007	**0.075**	0.040	0.026
economic situation	-0.014	-0.007	-0.015	-0.033	**-0.040**	0.012	-0.014	-0.025	**-0.058**	**-0.103**	0.024	**-0.052**	-0.005
drugs prescribed	**-0.056**	-0.015	-0.008	-0.023	**-0.070**	0.009	0.059	-0.023	0.007	-0.002	**-0.059**	**-0.081**	**0.096**
drugs non-prescribed	0.026	-0.007	0.020	-0.002	0.006	-0.003	-0.038	**0.065**	**0.052**	-0.014	-0.025	**0.045**	-0.013
practical doctor visits	0.022	0.009	0.012	0.025	0.027	**0.068**	0.018	0.004	0.024	**-0.058**	**-0.085**	**-0.100**	0.032
antibiotics	0.019	-0.017	0.028	0.032	0.000	0.021	-0.030	0.008	**0.066**	**-0.058**	**-0.098**	**-0.066**	-0.004
medical specialists visited	0.007	0.001	0.006	0.020	0.034	**0.056**	-0.006	**0.055**	**0.092**	-0.038	**-0.080**	**-0.074**	**0.045**
anxiety	0.021	**0.059**	-0.042	0.023	0.023	0.031	-0.009	**0.062**	**0.051**	**0.067**	-0.006	0.026	-0.024
phobia	-0.015	**0.050**	**-0.066**	0.021	0.030	**0.072**	-0.001	**0.060**	0.012	0.026	0.019	-0.009	-0.006
depression	-0.001	**0.047**	-0.040	0.035	**0.052**	**0.046**	-0.006	**0.072**	**0.064**	**0.071**	0.013	0.015	0.008
mania	0.020	**0.055**	-0.033	**0.069**	**0.057**	**0.098**	-0.072	**0.133**	**0.074**	**0.078**	**0.104**	**0.118**	**-0.046**
obsession	**-0.050**	**0.060**	**-0.098**	0.019	0.040	0.017	0.020	**0.085**	**0.085**	0.032	**0.047**	0.015	-0.008
audial halucination	0.026	-0.021	0.034	0.044	0.005	0.026	-0.008	**0.077**	**0.074**	**0.050**	0.030	**0.054**	-0.012
visual halucination	0.006	-0.038	0.023	0.039	-0.012	0.024	0.041	**0.056**	**0.055**	**0.051**	0.037	**0.059**	-0.037
headache	0.036	**0.059**	-0.035	0.023	0.034	0.034	-0.075	0.019	0.011	-0.043	-0.001	**-0.068**	**0.048**
subjective physical health problems	-0.016	0.042	**-0.054**	-0.030	0.015	-0.001	0.026	-0.004	0.007	**0.107**	-0.001	-0.035	**0.147**
subjective mental health problems	-0.028	**0.044**	**-0.054**	-0.034	0.040	0.002	-0.054	0.005	0.026	**0.085**	-0.027	**-0.061**	-0.006
diagnosed psychiatric disorders	**-0.058**	**0.068**	**-0.084**	**0.071**	**0.056**	**0.073**	0.031	**0.055**	**0.072**	**0.091**	**-0.072**	-0.003	-0.002
non-diagnosed psychiatric disorders	-0.015	**0.062**	**-0.052**	**0.053**	0.030	**0.042**	0.024	**0.098**	**0.063**	**0.112**	0.027	0.027	-0.025
psychiatric disorders total number	-0.034	**0.078**	**-0.079**	**0.075**	**0.048**	**0.066**	0.011	**0.098**	**0.079**	**0.117**	-0.014	0.024	-0.026
partner's diagnosed psychiatric disorders	-0.038	-0.013	-0.018	0.036	-0.017	0.022	0.043	-0.020	0.028	0.037	-0.008	**0.086**	-0.025
partner's non-diagnosed psychiatric disord.	**-0.050**	**0.051**	**-0.074**	**0.089**	**0.080**	**0.111**	**0.180**	**0.044**	0.005	**0.058**	-0.021	0.024	-0.009
partner's psychiatric disord. total number	**-0.056**	0.023	**-0.062**	**0.084**	**0.052**	**0.099**	**0.128**	0.012	0.030	**0.060**	-0.013	**0.076**	-0.020
mental health problems score	-0.001	**0.073**	**-0.068**	**0.043**	0.026	**0.046**	-0.001	**0.094**	**0.086**	**0.058**	0.007	0.001	-0.013
physical health problems score	0.009	0.002	0.008	0.016	0.008	**0.049**	0.001	0.036	**0.073**	**-0.048**	**-0.095**	**-0.080**	**0.053**
sexual activity	**0.045**	0.003	0.021	**0.111**	0.042	0.037	0.063	**0.077**	**0.047**	**0.204**	**0.163**	**0.139**	0.026
sexual desire	0.032	0.032	0.026	0.039	0.014	-0.024	-0.025	0.012	0.038	0.012	**0.060**	0.031	0.000

b) p-values of two-sided tests

	like dogs	like cats	refer dog	dog bit	cat ever	cat now	ats numbe	cat bit	scratch	smoking	alcohol	egal dru	BMI
WHOQOL-BREF health	0.363	0.112	0.404	0.911	0.300	0.296	0.425	0.178	0.036	0.012	0.671	0.594	0.030
WHOQOL-BREF psychological	0.117	0.924	0.375	0.130	0.077	0.515	0.188	0.969	0.318	0.043	0.930	0.430	0.376
WHOQOL-BREF social relationships	0.192	0.834	0.409	0.272	0.069	0.942	0.409	0.631	0.325	0.984	0.064	0.122	0.471
WHOQOL-BREF environment	0.027	0.065	0.002	0.162	0.451	0.005	0.180	0.348	0.843	0.134	0.148	0.814	0.021
WHOQOL-BREF total score	0.620	0.220	0.272	0.978	0.099	0.170	0.634	0.710	0.076	0.031	0.168	0.273	0.029
children	0.003	0.000	0.593	0.704	0.699	0.465	0.939	0.903	0.641	0.001	0.882	0.234	0.000
siblings	0.003	0.001	0.878	0.064	0.592	0.384	0.995	0.222	0.675	0.607	0.927	0.706	0.652
family situation	0.327	0.598	0.929	0.028	0.593	0.962	0.080	0.283	0.003	0.733	0.000	0.053	0.188
economic situation	0.484	0.722	0.449	0.100	0.046	0.556	0.724	0.218	0.004	0.000	0.255	0.012	0.809
drugs prescribed	0.008	0.464	0.710	0.270	0.001	0.656	0.176	0.280	0.737	0.913	0.005	0.000	0.000
drugs non-prescribed	0.216	0.726	0.349	0.920	0.766	0.893	0.379	0.002	0.014	0.510	0.235	0.032	0.541
practical doctor visits	0.295	0.671	0.574	0.230	0.200	0.001	0.672	0.832	0.260	0.006	0.000	0.000	0.127
antibiotics	0.358	0.418	0.190	0.129	0.987	0.321	0.496	0.720	0.002	0.005	0.000	0.002	0.861
medical specialists visited	0.756	0.959	0.787	0.350	0.107	0.007	0.898	0.009	0.000	0.073	0.000	0.000	0.031
anxiety	0.331	0.006	0.053	0.294	0.279	0.151	0.838	0.004	0.019	0.002	0.789	0.227	0.278
phobia	0.506	0.026	0.003	0.340	0.180	0.001	0.990	0.007	0.577	0.252	0.393	0.689	0.796
depression	0.950	0.034	0.071	0.110	0.019	0.034	0.895	0.001	0.004	0.001	0.550	0.488	0.707
mania	0.393	0.017	0.161	0.003	0.014	0.000	0.134	0.000	0.001	0.001	0.000	0.000	0.049
obsession	0.028	0.008	0.000	0.404	0.077	0.461	0.676	0.000	0.000	0.159	0.037	0.514	0.709
audial halucination	0.262	0.361	0.149	0.063	0.836	0.269	0.872	0.001	0.002	0.033	0.196	0.022	0.607
visual halucination	0.791	0.112	0.332	0.098	0.599	0.308	0.407	0.019	0.020	0.030	0.118	0.013	0.115
headache	0.102	0.008	0.115	0.293	0.126	0.122	0.101	0.380	0.627	0.051	0.946	0.002	0.030
subjective physical health problems	0.474	0.056	0.014	0.170	0.498	0.976	0.563	0.847	0.748	0.000	0.978	0.105	0.000
subjective mental health problems	0.197	0.044	0.014	0.122	0.070	0.911	0.228	0.820	0.236	0.000	0.224	0.005	0.788
diagnosed psychiatric disorders	0.006	0.001	0.000	0.001	0.007	0.001	0.472	0.009	0.001	0.000	0.001	0.888	0.939
non-diagnosed psychiatric disorders	0.489	0.003	0.015	0.011	0.154	0.045	0.574	0.000	0.003	0.000	0.203	0.204	0.233
psychiatric disorders total number	0.106	0.000	0.000	0.000	0.021	0.002	0.807	0.000	0.000	0.000	0.509	0.244	0.217
partner's diagnosed psychiatric disorders	0.070	0.548	0.405	0.087	0.418	0.287	0.319	0.335	0.184	0.075	0.690	0.000	0.230
partner's non-diagnosed psychiatric disord.	0.018	0.016	0.000	0.000	0.000	0.000	0.000	0.034	0.816	0.005	0.312	0.244	0.658
partner's psychiatric disord. total number	0.008	0.266	0.004	0.000	0.013	0.000	0.003	0.576	0.158	0.004	0.549	0.000	0.330
mental health problems score	0.947	0.000	0.001	0.041	0.221	0.028	0.980	0.000	0.000	0.006	0.730	0.949	0.540
physical health problems score	0.680	0.925	0.713	0.440	0.708	0.020	0.991	0.087	0.000	0.021	0.000	0.000	0.012
sexual activity	0.046	0.885	0.361	0.000	0.060	0.093	0.167	0.001	0.034	0.000	0.000	0.000	0.240
sexual desire	0.151	0.154	0.246	0.075	0.539	0.269	0.582	0.585	0.085	0.594	0.007	0.160	0.986
