# Supplementary material for: Friends with malefit. The effects of keeping dogs and cats, sustaining animal-related injuries and Toxoplasma infection on health and quality of life
Source: PLoS One. 2019 Nov 22;14(11):e0221988. doi: 10.1371/journal.pone.0221988 (PMC6874301; doi:10.1371/journal.pone.0221988)
Supplement: S6 Table — (PDF) [file pone.0221988.s021.pdf]

Table S6: Partial Kendall correlation (age, education, and urbanization controlled) between variables listed in the first raw and first column.

WOMEN WHO NEVER KEPT A DOG

a) Partial Kendall Tau (significant Tau printed bold, no correction for multiple comparission. Blue cells and red cells indicate negative and positive correlation, respectively.)

	like dogs	like cats	refer dog	dog bit	cat ever	cat now	ats numb	cat bit	scratch	smoking	alcohol	egal dru	BMI
WHOQOL-BREF health	0.018	-0.002	0.023	-0.009	**-0.044**	-0.025	0.008	**-0.056**	**-0.063**	-0.021	0.008	-0.026	**-0.076**
WHOQOL-BREF psychological	-0.017	0.000	-0.002	-0.015	-0.012	-0.023	-0.045	-0.026	-0.024	**-0.074**	-0.032	**-0.064**	**-0.074**
WHOQOL-BREF social relationships	0.010	0.031	-0.011	-0.034	-0.025	0.016	0.016	-0.022	-0.011	**-0.048**	0.004	-0.024	**-0.066**
WHOQOL-BREF environment	-0.006	0.014	-0.002	-0.021	-0.034	-0.006	-0.048	**-0.071**	**-0.057**	**-0.064**	**0.051**	-0.035	-0.033
WHOQOL-BREF total score	0.001	0.008	0.008	-0.022	-0.036	-0.017	-0.034	**-0.057**	**-0.056**	**-0.057**	0.020	**-0.052**	**-0.082**
children	**-0.128**	**-0.101**	-0.023	**0.051**	0.034	**-0.046**	-0.034	-0.027	**-0.058**	-0.034	**-0.044**	-0.026	**0.047**
siblings	**-0.062**	**-0.039**	-0.028	0.022	0.033	0.014	**0.071**	-0.001	-0.022	**-0.047**	-0.032	-0.026	-0.006
family situation	-0.003	-0.003	0.015	-0.026	**-0.041**	-0.003	0.017	0.000	0.000	**-0.082**	-0.005	-0.026	-0.034
economic situation	-0.005	**-0.073**	**0.059**	**-0.040**	**-0.072**	**-0.080**	-0.047	**-0.052**	-0.026	**-0.105**	**-0.037**	**-0.073**	-0.025
drugs prescribed	0.008	-0.003	0.011	-0.003	0.001	0.032	**0.073**	-0.007	0.019	0.009	**-0.094**	**-0.044**	**0.086**
drugs non-prescribed	**0.059**	0.033	0.008	0.008	-0.007	0.024	-0.016	-0.003	**0.037**	-0.002	-0.026	**0.056**	-0.011
practical doctor visits	**0.050**	**0.044**	-0.001	0.020	0.029	0.029	0.039	0.020	0.009	-0.008	0.004	0.030	**0.048**
antibiotics	**0.053**	0.024	0.028	0.032	0.021	0.006	**0.072**	0.026	**0.049**	**0.059**	**0.072**	**0.043**	0.034
medical specialists visited	0.027	0.002	0.021	0.029	-0.017	-0.016	0.045	0.002	0.029	0.016	-0.035	-0.015	0.026
anxiety	0.028	**0.046**	-0.019	0.034	0.035	**0.051**	0.013	**0.074**	**0.082**	**0.089**	**0.050**	**0.053**	-0.003
phobia	-0.036	**0.039**	**-0.061**	**0.051**	0.029	**0.051**	0.026	0.032	**0.049**	**0.046**	0.023	0.025	-0.001
depression	0.017	**0.053**	**-0.039**	0.035	**0.069**	**0.065**	0.052	**0.070**	**0.059**	**0.093**	**0.047**	**0.076**	0.036
mania	**-0.051**	-0.020	**-0.042**	0.015	0.031	0.038	0.048	0.007	**0.043**	**0.103**	**0.054**	**0.089**	0.005
obsession	-0.038	0.014	**-0.058**	0.029	0.001	0.034	0.050	0.020	**0.060**	**0.066**	0.020	0.036	-0.003
audial hallucination	-0.034	-0.012	-0.024	**0.048**	0.021	0.014	0.032	0.031	**0.064**	**0.051**	0.009	**0.087**	0.021
visual halucination	-0.021	-0.017	-0.024	**0.056**	0.027	0.024	0.022	**0.056**	**0.082**	**0.046**	-0.011	**0.057**	0.006
headache	-0.005	**0.064**	**-0.050**	0.035	0.032	**0.052**	0.022	**0.046**	**0.042**	**0.049**	**0.066**	**0.040**	0.023
subjective physical health problems	-0.010	0.037	-0.036	-0.035	0.026	**0.071**	0.035	0.005	0.016	**0.042**	**-0.074**	-0.016	**0.200**
subjective mental health problems	**0.038**	0.022	0.004	-0.030	0.019	**0.049**	0.029	0.013	0.020	**0.055**	-0.032	0.008	0.034
diagnosed psychiatric disorders	-0.013	**0.083**	**-0.085**	0.035	**0.103**	**0.113**	0.049	**0.079**	**0.082**	**0.120**	**-0.057**	0.013	**0.054**
non-diagnosed psychiatric disorders	-0.006	**0.085**	**-0.074**	0.008	**0.054**	**0.081**	0.000	**0.039**	0.009	**0.090**	**0.037**	**0.055**	0.020
psychiatric disorders total number	-0.008	**0.101**	**-0.091**	0.036	**0.085**	**0.116**	0.035	**0.069**	**0.041**	**0.129**	0.000	**0.053**	0.034
partner's diagnosed psychiatric disorders	-0.016	0.001	-0.022	0.028	**0.054**	0.020	**0.106**	-0.006	-0.015	**0.078**	0.023	0.010	0.004
partner's non-diagnosed psychiatric disord.	0.002	0.008	-0.001	-0.032	**0.049**	0.000	0.024	-0.011	0.002	**0.037**	-0.010	-0.005	**0.043**
partner's psychiatric disord. total number	-0.005	0.009	-0.014	0.004	**0.053**	0.009	**0.090**	-0.010	0.000	**0.059**	0.014	0.016	0.009
mental health problems score	-0.001	**0.079**	**-0.070**	**0.056**	**0.069**	**0.082**	0.060	**0.081**	**0.078**	**0.108**	**0.047**	**0.062**	0.023
physical health problems score	**0.066**	**0.042**	0.020	0.032	0.010	0.023	0.052	0.016	**0.037**	0.031	-0.020	0.023	**0.053**
sexual activity	-0.008	**0.062**	**-0.040**	**0.043**	**0.070**	0.025	0.014	**0.054**	-0.003	**0.241**	**0.174**	**0.187**	0.028
sexual desire	**0.061**	**0.090**	0.010	-0.001	0.002	-0.007	-0.016	0.000	0.020	-0.011	0.030	-0.007	-0.030

b) p-values of two-sided tests

	like dogs	like cats	refer dog	dog bit	cat ever	cat now	ats numb	cat bit	scratch	smoking	alcohol	egal dru	BMI
WHOQOL-BREF health	0.368	0.934	0.248	0.647	0.023	0.190	0.819	0.004	0.001	0.274	0.672	0.177	0.000
WHOQOL-BREF psychological	0.372	0.998	0.918	0.427	0.520	0.233	0.169	0.175	0.221	0.000	0.103	0.001	0.000
WHOQOL-BREF social relationships	0.618	0.112	0.578	0.076	0.196	0.407	0.621	0.251	0.579	0.012	0.826	0.209	0.001
WHOQOL-BREF environment	0.766	0.484	0.931	0.286	0.076	0.772	0.146	0.000	0.003	0.001	0.008	0.075	0.088
WHOQOL-BREF total score	0.951	0.695	0.701	0.262	0.064	0.386	0.312	0.004	0.005	0.004	0.297	0.008	0.000
children	0.000	0.000	0.192	0.004	0.053	0.009	0.262	0.130	0.001	0.065	0.016	0.164	0.007
siblings	0.000	0.025	0.115	0.220	0.057	0.424	0.018	0.975	0.218	0.011	0.080	0.163	0.713
family situation	0.883	0.857	0.394	0.144	0.019	0.884	0.584	0.991	0.998	0.000	0.785	0.166	0.055
economic situation	0.759	0.000	0.001	0.022	0.000	0.000	0.123	0.003	0.138	0.000	0.043	0.000	0.153
drugs prescribed	0.684	0.867	0.554	0.864	0.953	0.087	0.022	0.705	0.296	0.637	0.000	0.017	0.000
drugs non-prescribed	0.002	0.078	0.675	0.684	0.703	0.199	0.605	0.854	0.048	0.920	0.159	0.002	0.560
practical doctor visits	0.008	0.017	0.953	0.281	0.117	0.114	0.216	0.279	0.615	0.660	0.836	0.107	0.010
antibiotics	0.004	0.203	0.133	0.079	0.255	0.725	0.023	0.161	0.008	0.001	0.000	0.020	0.068
medical specialists visited	0.151	0.926	0.251	0.119	0.354	0.390	0.154	0.934	0.123	0.391	0.057	0.409	0.160
anxiety	0.139	0.015	0.305	0.071	0.066	0.007	0.693	0.000	0.000	0.000	0.008	0.005	0.891
phobia	0.060	0.040	0.002	0.008	0.123	0.007	0.434	0.091	0.011	0.017	0.238	0.196	0.955
depression	0.383	0.005	0.043	0.064	0.000	0.001	0.112	0.000	0.002	0.000	0.014	0.000	0.060
mania	0.009	0.310	0.033	0.436	0.121	0.052	0.155	0.724	0.031	0.000	0.006	0.000	0.786
obsession	0.054	0.485	0.003	0.139	0.962	0.085	0.131	0.308	0.002	0.001	0.319	0.070	0.891
audial hallucination	0.087	0.549	0.237	0.016	0.304	0.489	0.352	0.127	0.002	0.011	0.646	0.000	0.291
visual halucination	0.294	0.403	0.241	0.005	0.178	0.237	0.527	0.005	0.000	0.023	0.585	0.005	0.747
headache	0.776	0.001	0.009	0.064	0.094	0.006	0.506	0.015	0.025	0.009	0.000	0.032	0.232
subjective physical health problems	0.603	0.051	0.064	0.065	0.184	0.000	0.281	0.794	0.417	0.029	0.000	0.418	0.000
subjective mental health problems	0.047	0.252	0.828	0.116	0.325	0.010	0.385	0.496	0.295	0.004	0.094	0.662	0.078
diagnosed psychiatric disorders	0.499	0.000	0.000	0.062	0.000	0.000	0.124	0.000	0.000	0.000	0.002	0.468	0.004
non-diagnosed psychiatric disorders	0.732	0.000	0.000	0.665	0.003	0.000	0.988	0.034	0.635	0.000	0.043	0.003	0.273
psychiatric disorders total number	0.684	0.000	0.000	0.052	0.000	0.000	0.275	0.000	0.028	0.000	1.000	0.004	0.068
partner's diagnosed psychiatric disorders	0.384	0.954	0.246	0.130	0.004	0.272	0.001	0.736	0.413	0.000	0.209	0.582	0.816
partner's non-diagnosed psychiatric disord.	0.917	0.677	0.951	0.087	0.008	0.980	0.440	0.562	0.894	0.045	0.591	0.774	0.020
partner's psychiatric disord. total number	0.780	0.633	0.459	0.810	0.004	0.617	0.005	0.601	0.982	0.001	0.437	0.397	0.630
mental health problems score	0.948	0.000	0.000	0.002	0.000	0.000	0.055	0.000	0.000	0.000	0.011	0.001	0.210
physical health problems score	0.000	0.023	0.281	0.081	0.579	0.206	0.103	0.394	0.043	0.095	0.267	0.205	0.004
sexual activity	0.676	0.002	0.045	0.030	0.000	0.205	0.684	0.007	0.899	0.000	0.000	0.000	0.162
sexual desire	0.003	0.000	0.625	0.963	0.909	0.732	0.647	0.983	0.324	0.590	0.141	0.716	0.137
