# Supplementary material for: Friends with malefit. The effects of keeping dogs and cats, sustaining animal-related injuries and Toxoplasma infection on health and quality of life
Source: PLoS One. 2019 Nov 22;14(11):e0221988. doi: 10.1371/journal.pone.0221988 (PMC6874301; doi:10.1371/journal.pone.0221988)
Supplement: S7 Table — (PDF) [file pone.0221988.s022.pdf]

Table S7: Partial Kendall correlation (age, education, and urbanization controlled) between variables listed in the first raw and first column.

MEN WHO NEVER KEPT A CAT

**a) Partial Kendall Tau (significant Tau printed bold, no correction for multiple comparission. Blue cells and red cells indicate negative and positive correlation, respectively.)**

| | like dogs | like cats | refer dog | dog ever | dog now | ogs numb | dog bit | cat bit | scratch | smoking | alcohol | egal dru | BMI |
|---|---|---|---|---|---|---|---|---|---|---|---|---|---|
| WHOQOL-BREF health | 0.002 | 0.020 | -0.013 | -0.009 | -0.008 | 0.015 | -0.007 | -0.014 | -0.002 | -0.031 | 0.008 | -0.015 | **-0.075** |
| WHOQOL-BREF psychological | 0.034 | -0.009 | 0.032 | 0.027 | 0.010 | 0.014 | 0.002 | -0.022 | -0.008 | -0.030 | -0.014 | -0.037 | **-0.055** |
| WHOQOL-BREF social relationships | 0.020 | 0.012 | -0.001 | -0.006 | 0.011 | 0.015 | 0.035 | -0.031 | 0.001 | 0.028 | 0.031 | 0.017 | **-0.056** |
| WHOQOL-BREF environment | -0.001 | 0.034 | -0.041 | -0.023 | -0.007 | 0.015 | -0.037 | -0.034 | -0.039 | **-0.060** | **0.048** | -0.005 | -0.038 |
| WHOQOL-BREF total score | 0.015 | 0.018 | -0.010 | -0.003 | -0.006 | 0.007 | -0.006 | -0.033 | -0.017 | -0.036 | 0.017 | -0.009 | **-0.072** |
| children | **-0.070** | **-0.044** | -0.013 | 0.018 | -0.003 | 0.065 | -0.021 | -0.016 | -0.024 | 0.015 | -0.009 | **-0.064** | **0.088** |
| siblings | **-0.050** | -0.018 | **-0.040** | -0.015 | -0.004 | 0.006 | **-0.051** | **-0.051** | -0.010 | -0.025 | -0.023 | -0.037 | -0.031 |
| family situation | -0.007 | -0.023 | 0.011 | 0.007 | 0.031 | 0.004 | 0.022 | **-0.041** | **-0.052** | -0.007 | **0.047** | 0.022 | 0.006 |
| economic situation | -0.035 | -0.011 | -0.017 | **-0.042** | 0.001 | -0.050 | -0.012 | **-0.039** | -0.031 | **-0.103** | -0.007 | **-0.069** | 0.010 |
| drugs prescribed | -0.015 | -0.024 | -0.005 | -0.020 | 0.000 | **0.264** | -0.022 | -0.024 | -0.037 | 0.024 | **-0.056** | **-0.066** | **0.120** |
| drugs non-prescribed | **0.065** | -0.020 | **0.066** | **0.063** | **0.040** | -0.019 | 0.002 | 0.006 | 0.034 | -0.025 | -0.005 | 0.020 | -0.005 |
| practical doctor visits | 0.027 | -0.030 | **0.043** | 0.026 | 0.023 | **0.088** | -0.011 | -0.003 | 0.006 | -0.032 | **-0.043** | -0.029 | **0.046** |
| antibiotics | 0.004 | **-0.046** | 0.030 | -0.013 | 0.020 | **0.110** | -0.021 | -0.036 | -0.007 | **-0.049** | -0.003 | -0.022 | 0.025 |
| medical specialists visited | 0.010 | **-0.047** | **0.044** | -0.004 | **0.056** | 0.032 | 0.013 | 0.016 | 0.025 | -0.026 | -0.032 | -0.029 | **0.039** |
| anxiety | 0.025 | 0.032 | 0.001 | -0.012 | 0.005 | 0.072 | **0.050** | 0.032 | 0.033 | **0.075** | 0.018 | **0.101** | -0.010 |
| phobia | -0.006 | **0.046** | -0.034 | -0.008 | 0.032 | 0.035 | 0.027 | **0.059** | -0.001 | **0.043** | 0.029 | **0.063** | 0.014 |
| depression | 0.037 | 0.031 | 0.002 | 0.034 | 0.023 | 0.078 | **0.064** | **0.074** | 0.016 | **0.075** | 0.033 | **0.113** | 0.013 |
| mania | 0.004 | 0.028 | -0.004 | 0.007 | **0.051** | 0.069 | **0.078** | **0.104** | **0.045** | **0.091** | **0.094** | **0.181** | -0.033 |
| obsession | -0.016 | 0.017 | -0.024 | -0.039 | 0.000 | -0.055 | 0.033 | 0.033 | 0.027 | **0.042** | **0.067** | **0.100** | -0.008 |
| audial hallucination | -0.002 | 0.018 | -0.007 | -0.022 | **0.052** | -0.032 | 0.028 | **0.095** | **0.055** | **0.077** | **0.045** | **0.084** | -0.018 |
| visual halucination | -0.021 | 0.013 | -0.016 | -0.032 | 0.044 | -0.050 | 0.018 | **0.058** | 0.035 | 0.024 | **0.045** | **0.079** | **-0.048** |
| headache | 0.028 | 0.011 | 0.004 | 0.005 | 0.017 | -0.030 | 0.032 | -0.013 | 0.021 | -0.008 | -0.020 | -0.002 | **0.053** |
| subjective physical health problems | -0.002 | 0.004 | -0.018 | -0.002 | 0.001 | -0.004 | -0.038 | 0.003 | -0.012 | **0.085** | -0.021 | 0.001 | **0.189** |
| subjective mental health problems | 0.018 | 0.032 | -0.011 | 0.009 | **0.043** | 0.084 | -0.025 | -0.008 | -0.013 | **0.045** | -0.009 | 0.012 | 0.031 |
| diagnosed psychiatric disorders | 0.012 | 0.008 | 0.001 | **0.053** | 0.026 | **0.099** | **0.083** | 0.034 | **0.041** | **0.095** | **-0.052** | **0.044** | **0.054** |
| non-diagnosed psychiatric disorders | 0.037 | 0.021 | 0.002 | 0.031 | 0.003 | 0.039 | **0.064** | **0.092** | 0.026 | **0.074** | 0.032 | **0.128** | 0.035 |
| psychiatric disorders total number | 0.037 | 0.016 | 0.006 | **0.051** | 0.009 | **0.087** | **0.082** | **0.091** | **0.046** | **0.101** | -0.011 | **0.110** | **0.053** |
| partner's diagnosed psychiatric disorders | 0.009 | 0.000 | 0.002 | 0.021 | -0.010 | 0.006 | 0.022 | 0.034 | **0.054** | 0.009 | 0.020 | **0.085** | 0.004 |
| partner's non-diagnosed psychiatric disord. | 0.015 | -0.001 | 0.026 | **0.048** | **0.066** | 0.034 | 0.010 | -0.021 | 0.031 | -0.009 | -0.024 | 0.023 | **-0.043** |
| partner's psychiatric disord. total number | 0.012 | -0.006 | 0.015 | **0.047** | 0.019 | 0.027 | 0.028 | 0.015 | **0.058** | 0.002 | 0.003 | **0.079** | -0.014 |
| mental health problems score | 0.024 | 0.039 | -0.015 | -0.005 | 0.017 | 0.063 | **0.060** | **0.074** | **0.049** | **0.077** | 0.010 | **0.109** | 0.012 |
| physical health problems score | 0.032 | **-0.043** | **0.054** | 0.024 | **0.042** | **0.135** | -0.002 | -0.005 | 0.009 | -0.024 | **-0.047** | -0.032 | **0.062** |
| sexual activity | **0.091** | -0.038 | **0.101** | **0.102** | 0.014 | 0.009 | **0.121** | **0.069** | **0.062** | **0.209** | **0.102** | **0.155** | 0.037 |
| sexual desire | **0.073** | 0.019 | **0.050** | 0.037 | 0.034 | 0.003 | **0.044** | 0.003 | -0.008 | 0.015 | 0.030 | 0.034 | 0.004 |

**b) p-values of two-sided tests**

| | like dogs | like cats | refer dog | dog ever | dog now | ogs numb | dog bit | cat bit | scratch | smoking | alcohol | egal dru | BMI |
|---|---|---|---|---|---|---|---|---|---|---|---|---|---|
| WHOQOL-BREF health | 0.936 | 0.353 | 0.546 | 0.680 | 0.707 | 0.733 | 0.753 | 0.521 | 0.941 | 0.138 | 0.707 | 0.464 | 0.000 |
| WHOQOL-BREF psychological | 0.103 | 0.686 | 0.128 | 0.202 | 0.617 | 0.753 | 0.941 | 0.290 | 0.716 | 0.158 | 0.518 | 0.073 | 0.009 |
| WHOQOL-BREF social relationships | 0.348 | 0.561 | 0.966 | 0.788 | 0.615 | 0.731 | 0.092 | 0.146 | 0.956 | 0.174 | 0.141 | 0.415 | 0.008 |
| WHOQOL-BREF environment | 0.952 | 0.112 | 0.055 | 0.281 | 0.730 | 0.735 | 0.074 | 0.103 | 0.065 | 0.004 | 0.021 | 0.822 | 0.069 |
| WHOQOL-BREF total score | 0.486 | 0.399 | 0.655 | 0.888 | 0.769 | 0.877 | 0.782 | 0.125 | 0.422 | 0.091 | 0.418 | 0.673 | 0.001 |
| children | 0.000 | 0.021 | 0.498 | 0.341 | 0.892 | 0.092 | 0.268 | 0.407 | 0.215 | 0.446 | 0.666 | 0.001 | 0.000 |
| siblings | 0.009 | 0.355 | 0.036 | 0.440 | 0.825 | 0.874 | 0.007 | 0.007 | 0.588 | 0.215 | 0.247 | 0.066 | 0.097 |
| family situation | 0.700 | 0.233 | 0.565 | 0.718 | 0.104 | 0.909 | 0.239 | 0.034 | 0.006 | 0.712 | 0.018 | 0.269 | 0.742 |
| economic situation | 0.063 | 0.571 | 0.376 | 0.025 | 0.944 | 0.193 | 0.511 | 0.042 | 0.105 | 0.000 | 0.728 | 0.001 | 0.593 |
| drugs prescribed | 0.467 | 0.227 | 0.806 | 0.311 | 0.990 | 0.000 | 0.266 | 0.236 | 0.066 | 0.228 | 0.005 | 0.001 | 0.000 |
| drugs non-prescribed | 0.001 | 0.330 | 0.001 | 0.002 | 0.043 | 0.639 | 0.934 | 0.765 | 0.091 | 0.205 | 0.789 | 0.310 | 0.809 |
| practical doctor visits | 0.175 | 0.143 | 0.033 | 0.195 | 0.245 | 0.034 | 0.592 | 0.869 | 0.754 | 0.107 | 0.031 | 0.141 | 0.021 |
| antibiotics | 0.847 | 0.023 | 0.141 | 0.506 | 0.330 | 0.008 | 0.302 | 0.077 | 0.717 | 0.014 | 0.886 | 0.267 | 0.214 |
| medical specialists visited | 0.614 | 0.021 | 0.029 | 0.855 | 0.005 | 0.445 | 0.501 | 0.436 | 0.213 | 0.199 | 0.113 | 0.144 | 0.049 |
| anxiety | 0.227 | 0.125 | 0.967 | 0.559 | 0.816 | 0.097 | 0.014 | 0.127 | 0.111 | 0.000 | 0.368 | 0.000 | 0.619 |
| phobia | 0.766 | 0.033 | 0.109 | 0.691 | 0.127 | 0.434 | 0.196 | 0.006 | 0.972 | 0.042 | 0.174 | 0.003 | 0.503 |
| depression | 0.072 | 0.143 | 0.936 | 0.101 | 0.272 | 0.073 | 0.002 | 0.000 | 0.436 | 0.000 | 0.108 | 0.000 | 0.527 |
| mania | 0.846 | 0.198 | 0.840 | 0.743 | 0.019 | 0.135 | 0.000 | 0.000 | 0.040 | 0.000 | 0.000 | 0.000 | 0.128 |
| obsession | 0.469 | 0.447 | 0.268 | 0.073 | 0.992 | 0.232 | 0.120 | 0.132 | 0.223 | 0.050 | 0.002 | 0.000 | 0.723 |
| audial hallucination | 0.935 | 0.426 | 0.757 | 0.336 | 0.020 | 0.499 | 0.213 | 0.000 | 0.015 | 0.001 | 0.045 | 0.000 | 0.433 |
| visual halucination | 0.358 | 0.557 | 0.485 | 0.154 | 0.051 | 0.309 | 0.423 | 0.011 | 0.131 | 0.284 | 0.049 | 0.001 | 0.036 |
| headache | 0.182 | 0.606 | 0.848 | 0.805 | 0.412 | 0.500 | 0.132 | 0.524 | 0.311 | 0.698 | 0.344 | 0.922 | 0.012 |
| subjective physical health problems | 0.934 | 0.840 | 0.396 | 0.930 | 0.959 | 0.923 | 0.071 | 0.880 | 0.575 | 0.000 | 0.302 | 0.978 | 0.000 |
| subjective mental health problems | 0.402 | 0.129 | 0.597 | 0.677 | 0.036 | 0.057 | 0.229 | 0.689 | 0.541 | 0.032 | 0.663 | 0.555 | 0.143 |
| diagnosed psychiatric disorders | 0.548 | 0.682 | 0.953 | 0.008 | 0.193 | 0.018 | 0.000 | 0.094 | 0.041 | 0.000 | 0.009 | 0.028 | 0.006 |
| non-diagnosed psychiatric disorders | 0.067 | 0.290 | 0.908 | 0.124 | 0.896 | 0.342 | 0.001 | 0.000 | 0.198 | 0.000 | 0.108 | 0.000 | 0.084 |
| psychiatric disorders total number | 0.065 | 0.441 | 0.763 | 0.010 | 0.664 | 0.036 | 0.000 | 0.000 | 0.023 | 0.000 | 0.588 | 0.000 | 0.008 |
| partner's diagnosed psychiatric disorders | 0.664 | 0.999 | 0.920 | 0.283 | 0.615 | 0.886 | 0.265 | 0.087 | 0.007 | 0.641 | 0.319 | 0.000 | 0.826 |
| partner's non-diagnosed psychiatric disord. | 0.453 | 0.963 | 0.194 | 0.015 | 0.001 | 0.414 | 0.633 | 0.297 | 0.119 | 0.657 | 0.233 | 0.245 | 0.031 |
| partner's psychiatric disord. total number | 0.551 | 0.761 | 0.448 | 0.017 | 0.337 | 0.520 | 0.157 | 0.451 | 0.004 | 0.901 | 0.881 | 0.000 | 0.480 |
| mental health problems score | 0.236 | 0.053 | 0.450 | 0.805 | 0.390 | 0.126 | 0.002 | 0.000 | 0.015 | 0.000 | 0.613 | 0.000 | 0.548 |
| physical health problems score | 0.109 | 0.032 | 0.008 | 0.232 | 0.035 | 0.001 | 0.935 | 0.821 | 0.663 | 0.225 | 0.019 | 0.104 | 0.002 |
| sexual activity | 0.000 | 0.081 | 0.000 | 0.000 | 0.512 | 0.842 | 0.000 | 0.001 | 0.004 | 0.000 | 0.000 | 0.000 | 0.082 |
| sexual desire | 0.001 | 0.383 | 0.019 | 0.076 | 0.110 | 0.952 | 0.036 | 0.870 | 0.724 | 0.481 | 0.155 | 0.110 | 0.861 |
