# Supplementary material for: Friends with malefit. The effects of keeping dogs and cats, sustaining animal-related injuries and Toxoplasma infection on health and quality of life
Source: PLoS One. 2019 Nov 22;14(11):e0221988. doi: 10.1371/journal.pone.0221988 (PMC6874301; doi:10.1371/journal.pone.0221988)
Supplement: S8 Table — (PDF) [file pone.0221988.s023.pdf]

Table S8: Partial Kendall correlation (age, education, and urbanization controlled) between variables listed in the first raw and first column

WOMEN WHO NEVER KEPT A CAT

a) Partial Kendall Tau (significant Tau printed bold, no correction for multiple comparission. Blue cells and red cells indicate negative and positive correlation, respectively.)

	like dogs	like cats	refer dog	dog ever	dog now	ogs numb	dog bit	cat bit	scratch	smoking	alcohol	egal dru	BMI
WHOQOL-BREF health	0.019	0.018	-0.010	-0.006	-0.019	-0.022	**-0.041**	-0.012	**-0.064**	-0.025	0.032	-0.012	**-0.058**
WHOQOL-BREF psychological	-0.013	-0.010	-0.013	0.004	-0.001	0.011	**-0.051**	-0.015	-0.035	**-0.052**	-0.026	**-0.050**	**-0.049**
WHOQOL-BREF social relationships	0.000	**0.049**	**-0.053**	-0.026	-0.003	0.005	**-0.048**	0.002	-0.023	**-0.052**	-0.003	-0.020	-0.035
WHOQOL-BREF environment	-0.003	0.004	-0.015	**-0.037**	-0.012	0.010	**-0.036**	0.000	-0.010	-0.021	**0.053**	-0.001	-0.026
WHOQOL-BREF total score	-0.002	0.011	-0.021	-0.015	-0.010	0.000	**-0.056**	-0.006	**-0.045**	**-0.044**	0.023	-0.024	**-0.055**
children	**-0.117**	**-0.109**	0.019	0.022	**-0.046**	-0.025	0.011	**-0.038**	**-0.037**	**-0.053**	**-0.056**	-0.010	**0.050**
siblings	**-0.066**	-0.021	-0.023	-0.010	-0.030	0.019	0.003	-0.001	0.009	-0.029	-0.020	0.024	-0.015
family situation	-0.027	-0.002	**-0.035**	**-0.056**	-0.014	-0.033	**-0.051**	-0.029	0.005	**-0.059**	-0.016	-0.029	-0.031
economic situation	**-0.054**	-0.022	-0.030	**-0.052**	**-0.050**	-0.027	-0.024	**-0.048**	-0.014	**-0.055**	**-0.038**	-0.028	**-0.039**
drugs prescribed	-0.025	**-0.043**	0.027	0.002	-0.014	0.013	0.002	-0.009	0.022	**-0.051**	**-0.093**	**-0.061**	**0.070**
drugs non-prescribed	0.023	0.010	0.019	0.002	0.001	-0.002	0.008	-0.030	-0.010	-0.017	-0.013	**0.062**	-0.017
practical doctor visits	0.030	0.005	0.012	0.005	-0.005	-0.034	0.001	0.017	0.025	**-0.062**	-0.025	0.001	0.026
antibiotics	0.032	-0.005	**0.037**	0.026	0.016	0.052	0.032	**0.038**	0.033	0.012	0.021	0.017	0.021
medical specialists visited	-0.014	-0.020	0.010	-0.010	0.007	-0.014	**0.041**	0.021	**0.074**	-0.005	**-0.036**	-0.013	0.032
anxiety	-0.005	0.029	-0.007	0.030	0.012	0.001	**0.071**	**0.065**	**0.086**	**0.050**	0.034	0.033	-0.008
phobia	-0.013	0.020	-0.023	0.022	0.002	-0.017	**0.060**	0.015	0.034	0.002	0.001	-0.024	-0.003
depression	0.014	0.019	0.007	**0.051**	0.016	0.050	**0.075**	**0.079**	**0.079**	**0.071**	**0.052**	**0.054**	0.017
mania	**-0.041**	0.017	-0.026	**0.048**	-0.003	-0.057	**0.043**	**0.061**	**0.054**	**0.076**	**0.048**	**0.099**	0.018
obsession	**-0.037**	-0.006	-0.007	**0.039**	0.001	-0.025	0.026	**0.074**	**0.074**	**0.038**	**0.045**	**0.040**	-0.007
audial hallucination	-0.026	0.030	-0.023	**0.039**	0.024	0.042	**0.080**	**0.071**	**0.064**	**0.061**	0.022	**0.077**	0.030
visual halucination	-0.034	0.003	-0.014	0.034	0.024	-0.021	**0.069**	**0.065**	**0.060**	**0.048**	0.011	**0.064**	**0.039**
headache	-0.001	0.015	-0.005	0.032	0.030	**0.069**	0.030	0.024	**0.046**	0.005	0.022	0.014	0.011
subjective physical health problems	-0.001	-0.005	-0.017	-0.007	0.000	-0.004	-0.005	-0.023	0.026	**0.051**	**-0.056**	-0.010	**0.190**
subjective mental health problems	0.024	-0.001	0.018	0.017	0.008	0.054	0.027	0.010	0.022	**0.044**	-0.001	0.019	0.011
diagnosed psychiatric disorders	**0.039**	-0.003	0.016	**0.086**	**0.062**	0.013	**0.059**	**0.052**	**0.051**	**0.084**	**-0.034**	-0.010	0.034
non-diagnosed psychiatric disorders	0.009	0.015	-0.008	0.009	0.014	-0.001	**0.048**	**0.044**	0.019	**0.089**	**0.040**	0.033	0.002
psychiatric disorders total number	0.028	0.007	0.004	**0.049**	**0.039**	0.013	**0.074**	**0.065**	**0.040**	**0.122**	0.006	0.021	0.019
partner's diagnosed psychiatric disorders	0.008	-0.019	0.027	**0.055**	**0.042**	0.007	0.022	0.012	-0.028	**0.049**	0.028	**0.036**	0.013
partner's non-diagnosed psychiatric disord.	0.021	0.006	0.006	0.010	-0.006	-0.041	**-0.037**	**0.049**	0.000	0.009	0.004	-0.007	**0.046**
partner's psychiatric disord. total number	0.013	-0.010	0.020	0.034	0.013	-0.014	-0.004	0.020	-0.013	**0.045**	0.028	0.030	0.011
mental health problems score	0.000	0.023	-0.008	**0.046**	0.028	0.030	**0.073**	**0.075**	**0.081**	**0.087**	0.027	**0.043**	0.016
physical health problems score	0.015	-0.011	0.027	0.005	-0.004	-0.002	0.025	0.015	**0.040**	-0.027	**-0.038**	-0.001	**0.036**
sexual activity	**0.072**	-0.005	**0.051**	**0.100**	**0.056**	0.020	**0.046**	**0.079**	**0.044**	**0.253**	**0.162**	**0.139**	0.006
sexual desire	**0.126**	-0.015	**0.077**	0.020	0.014	0.050	0.008	-0.021	**-0.044**	**0.087**	**0.039**	**0.039**	-0.015

b) p-values of two-sided tests

	like dogs	like cats	refer dog	dog ever	dog now	ogs numb	dog bit	cat bit	scratch	smoking	alcohol	egal dru	BMI
WHOQOL-BREF health	0.296	0.339	0.567	0.746	0.294	0.501	0.025	0.528	0.000	0.164	0.079	0.512	0.001
WHOQOL-BREF psychological	0.478	0.598	0.491	0.843	0.975	0.729	0.005	0.398	0.059	0.004	0.144	0.006	0.007
WHOQOL-BREF social relationships	0.991	0.008	0.004	0.157	0.857	0.873	0.009	0.931	0.217	0.004	0.878	0.260	0.057
WHOQOL-BREF environment	0.856	0.818	0.421	0.039	0.520	0.758	0.050	0.983	0.571	0.255	0.003	0.946	0.160
WHOQOL-BREF total score	0.921	0.539	0.261	0.430	0.601	0.992	0.003	0.759	0.015	0.015	0.201	0.185	0.003
children	0.000	0.000	0.261	0.182	0.004	0.374	0.496	0.021	0.024	0.002	0.001	0.560	0.002
siblings	0.000	0.210	0.161	0.525	0.069	0.497	0.831	0.951	0.583	0.088	0.248	0.155	0.366
family situation	0.102	0.909	0.036	0.001	0.394	0.244	0.002	0.082	0.773	0.001	0.351	0.091	0.059
economic situation	0.001	0.185	0.068	0.001	0.002	0.352	0.144	0.003	0.408	0.001	0.028	0.098	0.016
drugs prescribed	0.155	0.013	0.116	0.899	0.410	0.667	0.890	0.585	0.215	0.003	0.000	0.000	0.000
drugs non-prescribed	0.175	0.567	0.266	0.917	0.949	0.953	0.627	0.087	0.548	0.321	0.466	0.000	0.316
practical doctor visits	0.078	0.769	0.480	0.754	0.751	0.264	0.953	0.317	0.151	0.000	0.155	0.963	0.135
antibiotics	0.065	0.756	0.036	0.129	0.362	0.091	0.065	0.030	0.063	0.471	0.231	0.337	0.225
medical specialists visited	0.403	0.253	0.555	0.581	0.664	0.648	0.017	0.237	0.000	0.762	0.039	0.443	0.064
anxiety	0.783	0.106	0.681	0.090	0.495	0.978	0.000	0.000	0.000	0.004	0.053	0.059	0.671
phobia	0.454	0.277	0.202	0.219	0.891	0.603	0.001	0.410	0.059	0.909	0.963	0.186	0.852
depression	0.434	0.284	0.681	0.004	0.357	0.114	0.000	0.000	0.000	0.000	0.004	0.002	0.344
mania	0.029	0.377	0.160	0.009	0.858	0.087	0.020	0.001	0.004	0.000	0.010	0.000	0.345
obsession	0.046	0.760	0.703	0.034	0.944	0.453	0.156	0.000	0.000	0.037	0.015	0.031	0.706
audial hallucination	0.175	0.121	0.234	0.044	0.217	0.220	0.000	0.000	0.001	0.002	0.251	0.000	0.121
visual halucination	0.077	0.885	0.466	0.078	0.215	0.536	0.000	0.001	0.002	0.012	0.570	0.001	0.041
headache	0.944	0.388	0.787	0.070	0.086	0.028	0.093	0.173	0.010	0.769	0.216	0.436	0.519
subjective physical health problems	0.959	0.780	0.355	0.697	0.985	0.894	0.788	0.211	0.144	0.005	0.002	0.561	0.000
subjective mental health problems	0.189	0.959	0.314	0.342	0.637	0.091	0.140	0.597	0.225	0.014	0.973	0.288	0.527
diagnosed psychiatric disorders	0.024	0.851	0.344	0.000	0.000	0.672	0.001	0.003	0.003	0.000	0.049	0.546	0.051
non-diagnosed psychiatric disorders	0.612	0.391	0.655	0.586	0.420	0.973	0.005	0.011	0.269	0.000	0.019	0.052	0.907
psychiatric disorders total number	0.101	0.686	0.813	0.005	0.025	0.675	0.000	0.000	0.023	0.000	0.717	0.215	0.278
partner's diagnosed psychiatric disorders	0.647	0.285	0.115	0.001	0.014	0.814	0.211	0.502	0.105	0.005	0.110	0.035	0.444
partner's non-diagnosed psychiatric disord.	0.228	0.724	0.712	0.576	0.731	0.173	0.032	0.005	0.988	0.586	0.799	0.693	0.009
partner's psychiatric disord. total number	0.436	0.585	0.263	0.052	0.439	0.638	0.839	0.238	0.450	0.010	0.111	0.081	0.510
mental health problems score	0.980	0.181	0.647	0.008	0.110	0.315	0.000	0.000	0.000	0.000	0.112	0.013	0.369
physical health problems score	0.397	0.535	0.125	0.751	0.823	0.949	0.140	0.379	0.022	0.121	0.026	0.954	0.039
sexual activity	0.000	0.789	0.007	0.000	0.003	0.554	0.014	0.000	0.020	0.000	0.000	0.000	0.752
sexual desire	0.000	0.450	0.000	0.297	0.452	0.141	0.673	0.267	0.022	0.000	0.041	0.042	0.437
