# Supplementary material for: Friends with malefit. The effects of keeping dogs and cats, sustaining animal-related injuries and Toxoplasma infection on health and quality of life
Source: PLoS One. 2019 Nov 22;14(11):e0221988. doi: 10.1371/journal.pone.0221988 (PMC6874301; doi:10.1371/journal.pone.0221988)
Supplement: S9 Table — (PDF) [file pone.0221988.s024.pdf]

Table S9: Partial Kendall correlation (age, education, and urbanization controlled) between variables listed in the first raw and first column.

MEN WHO WERE NEVER INJURED BY A DOG

a) Partial Kendall Tau (significant Tau printed bold, no correction for multiple comparission. Blue cells and red cells indicate negative and positive correlation, respectively.)

	like dogs	like cats	refer dog	dog ever	dog now	ogs numb	dog bit	cat ever	cat now	ats numb	cat bit	scratch	smoking	alcohol	egal dru	BMI
WHOQOL-BREF health	**0.049**	0.008	0.026	0.021	0.017	**-0.082**	-0.012	-0.006	-0.002	0.032	-0.024	**-0.035**	**-0.038**	0.020	**-0.037**	**-0.062**
WHOQOL-BREF psychological	**0.104**	-0.005	**0.069**	**0.051**	0.020	-0.012	**0.034**	-0.021	-0.009	-0.036	**-0.046**	**-0.049**	**-0.047**	0.001	**-0.063**	-0.030
WHOQOL-BREF social relationships	**0.075**	-0.007	**0.045**	0.030	**0.046**	**0.064**	**0.035**	0.008	0.007	-0.047	**-0.049**	**-0.043**	-0.023	0.027	-0.001	-0.024
WHOQOL-BREF environment	0.016	**0.036**	-0.021	-0.020	-0.010	-0.052	-0.002	-0.018	0.002	-0.017	-0.016	**-0.034**	**-0.065**	**0.051**	-0.016	**-0.044**
WHOQOL-BREF total score	**0.075**	0.008	**0.038**	0.019	0.009	-0.047	0.017	-0.015	-0.008	-0.031	**-0.040**	**-0.044**	**-0.058**	**0.034**	**-0.038**	**-0.054**
children	**-0.046**	**-0.101**	**0.048**	**0.037**	0.024	0.042	0.015	0.012	-0.020	-0.018	**-0.040**	-0.021	0.022	0.018	**-0.062**	**0.097**
siblings	**-0.038**	**-0.036**	-0.006	**0.045**	0.008	0.049	0.007	**0.034**	**0.040**	0.042	0.012	0.019	-0.017	-0.024	-0.020	-0.009
family situation	**0.054**	-0.020	**0.049**	0.015	**0.038**	0.019	0.027	0.007	-0.020	**-0.057**	**-0.050**	**-0.077**	**-0.037**	**0.047**	-0.024	0.014
economic situation	-0.008	-0.023	0.014	**-0.038**	**-0.030**	-0.037	**-0.037**	-0.023	**-0.041**	0.012	-0.026	-0.010	**-0.118**	0.014	**-0.085**	0.004
drugs prescribed	-0.009	-0.025	0.005	-0.008	0.014	**0.155**	**-0.045**	**-0.048**	-0.023	0.040	-0.014	0.006	-0.018	**-0.049**	**-0.053**	**0.125**
drugs non-prescribed	0.019	0.020	-0.001	**0.040**	0.002	0.007	**0.041**	-0.003	-0.003	0.012	0.018	**0.038**	-0.028	-0.004	**0.068**	-0.008
practical doctor visits	0.012	**-0.030**	**0.030**	0.002	-0.002	**0.128**	0.023	**-0.032**	0.004	0.000	0.009	0.013	**-0.051**	**-0.054**	**-0.042**	**0.061**
antibiotics	-0.022	**-0.043**	0.015	-0.007	0.003	**0.095**	0.028	-0.025	0.007	0.013	-0.018	0.011	-0.025	**-0.031**	-0.013	0.026
medical specialists visited	-0.027	-0.018	-0.010	-0.025	**0.034**	0.050	0.023	0.006	0.020	0.018	**0.036**	**0.069**	**-0.048**	**-0.038**	-0.016	0.026
anxiety	**-0.048**	**0.035**	**-0.061**	-0.020	0.011	-0.011	0.003	0.020	**0.036**	-0.043	**0.072**	**0.070**	**0.045**	0.020	**0.084**	**-0.036**
phobia	**-0.053**	0.027	**-0.057**	-0.022	0.010	0.025	-0.011	-0.002	**0.033**	0.003	**0.057**	**0.040**	-0.005	0.015	0.028	-0.016
depression	**-0.038**	**0.035**	**-0.054**	0.012	**0.030**	-0.014	0.006	**0.043**	**0.048**	0.011	**0.088**	**0.062**	**0.048**	0.029	**0.081**	0.004
mania	-0.029	**0.046**	**-0.049**	-0.002	0.008	-0.010	**0.064**	**0.035**	**0.060**	0.000	**0.097**	**0.058**	**0.039**	**0.048**	**0.129**	**-0.057**
obsession	**-0.067**	0.023	**-0.063**	-0.024	-0.010	-0.028	-0.027	**0.031**	0.020	0.043	**0.042**	**0.063**	-0.005	**0.034**	**0.055**	**-0.038**
audial hallucination	**-0.035**	-0.005	-0.016	0.007	0.016	-0.030	0.025	0.008	0.016	-0.049	0.030	0.018	0.018	0.011	**0.077**	-0.007
visual halucination	**-0.043**	-0.008	-0.019	0.000	0.013	-0.018	0.024	-0.001	0.009	0.009	**0.037**	0.017	0.018	0.013	**0.052**	-0.027
headache	-0.013	0.012	-0.029	-0.010	0.000	-0.014	0.011	0.016	0.020	0.000	**0.036**	**0.048**	-0.027	-0.026	-0.008	0.015
subjective physical health problems	**-0.039**	0.000	**-0.032**	-0.028	0.012	0.030	**-0.031**	-0.025	-0.016	-0.011	0.004	0.021	**0.080**	-0.027	**-0.050**	**0.163**
subjective mental health problems	**-0.073**	0.008	**-0.056**	**-0.044**	-0.012	-0.024	-0.014	-0.023	-0.019	-0.016	0.024	0.014	**0.035**	0.009	0.002	0.015
diagnosed psychiatric disorders	-0.013	**0.051**	**-0.048**	**0.032**	0.013	-0.008	0.026	**0.046**	**0.047**	**0.054**	**0.086**	**0.068**	**0.098**	-0.013	**0.059**	0.007
non-diagnosed psychiatric disorders	-0.020	**0.038**	**-0.043**	0.001	-0.013	-0.033	0.026	0.020	0.018	0.039	**0.080**	**0.055**	**0.090**	0.028	**0.077**	0.001
psychiatric disorders total number	-0.024	**0.053**	**-0.059**	0.016	-0.008	-0.035	0.025	**0.031**	**0.046**	0.050	**0.102**	**0.078**	**0.111**	0.015	**0.085**	0.008
partner's diagnosed psychiatric disorders	-0.002	0.006	-0.002	**0.043**	0.000	-0.011	0.019	0.012	**0.040**	0.024	0.027	**0.062**	0.000	0.007	**0.070**	0.008
partner's non-diagnosed psychiatric disord.	**0.031**	0.003	0.021	**0.039**	**0.054**	0.019	-0.003	0.027	**0.055**	**0.107**	0.003	0.022	**0.030**	0.007	**0.042**	0.002
partner's psychiatric disord. total number	0.012	0.000	0.008	**0.049**	0.022	-0.031	0.010	0.019	**0.056**	**0.069**	0.016	**0.056**	0.015	0.020	**0.069**	0.005
mental health problems score	**-0.039**	**0.053**	**-0.075**	-0.010	0.012	-0.029	0.004	0.025	**0.054**	0.011	**0.107**	**0.086**	**0.047**	0.010	**0.084**	-0.013
physical health problems score	0.004	-0.020	0.013	0.007	0.012	**0.121**	0.017	-0.021	0.004	0.003	0.016	**0.048**	**-0.040**	**-0.053**	-0.007	**0.059**
sexual activity	**0.084**	-0.006	**0.054**	**0.112**	0.021	0.036	**0.069**	**0.066**	0.015	-0.002	**0.046**	**0.035**	**0.215**	**0.122**	**0.159**	**0.050**
sexual desire	**0.086**	0.012	**0.057**	**0.036**	**0.041**	0.023	-0.013	0.021	-0.022	0.034	-0.003	-0.004	0.004	**0.033**	-0.029	0.028

b) p-values of two-sided tests

	like dogs	like cats	refer dog	dog ever	dog now	ogs numb	dog bit	cat ever	cat now	ats numb	cat bit	scratch	smoking	alcohol	egal dru	BMI
WHOQOL-BREF health	0.002	0.591	0.098	0.176	0.275	0.004	0.420	0.688	0.919	0.251	0.121	0.025	0.013	0.192	0.017	0.000
WHOQOL-BREF psychological	0.000	0.722	0.000	0.001	0.195	0.684	0.028	0.176	0.548	0.197	0.002	0.001	0.002	0.948	0.000	0.053
WHOQOL-BREF social relationships	0.000	0.635	0.003	0.052	0.003	0.026	0.021	0.620	0.655	0.087	0.001	0.005	0.132	0.077	0.930	0.121
WHOQOL-BREF environment	0.299	0.021	0.173	0.199	0.498	0.071	0.913	0.249	0.893	0.552	0.289	0.025	0.000	0.001	0.291	0.004
WHOQOL-BREF total score	0.000	0.628	0.017	0.218	0.557	0.108	0.272	0.335	0.631	0.283	0.011	0.005	0.000	0.032	0.017	0.001
children	0.001	0.000	0.001	0.008	0.080	0.095	0.273	0.369	0.155	0.463	0.004	0.131	0.130	0.212	0.000	0.000
siblings	0.007	0.010	0.673	0.001	0.563	0.053	0.598	0.014	0.004	0.096	0.395	0.175	0.246	0.107	0.174	0.531
family situation	0.000	0.142	0.000	0.286	0.006	0.464	0.052	0.613	0.155	0.025	0.000	0.000	0.013	0.001	0.097	0.315
economic situation	0.575	0.105	0.322	0.007	0.032	0.141	0.008	0.102	0.003	0.643	0.066	0.478	0.000	0.352	0.000	0.794
drugs prescribed	0.554	0.087	0.728	0.590	0.343	0.000	0.002	0.001	0.116	0.140	0.327	0.701	0.215	0.001	0.000	0.000
drugs non-prescribed	0.210	0.177	0.925	0.007	0.888	0.785	0.006	0.822	0.854	0.664	0.218	0.010	0.062	0.801	0.000	0.604
practical doctor visits	0.430	0.046	0.043	0.868	0.907	0.000	0.111	0.028	0.777	0.996	0.556	0.384	0.001	0.000	0.004	0.000
antibiotics	0.140	0.003	0.302	0.623	0.862	0.001	0.055	0.086	0.613	0.626	0.230	0.474	0.087	0.033	0.374	0.080
medical specialists visited	0.069	0.226	0.518	0.085	0.020	0.065	0.116	0.701	0.183	0.508	0.014	0.000	0.001	0.011	0.281	0.079
anxiety	0.002	0.022	0.000	0.193	0.466	0.695	0.821	0.200	0.018	0.124	0.000	0.000	0.003	0.192	0.000	0.018
phobia	0.001	0.084	0.000	0.155	0.530	0.397	0.494	0.915	0.038	0.907	0.000	0.010	0.730	0.342	0.070	0.299
depression	0.014	0.022	0.000	0.448	0.050	0.628	0.691	0.006	0.002	0.708	0.000	0.000	0.002	0.062	0.000	0.798
mania	0.069	0.005	0.002	0.881	0.632	0.748	0.000	0.029	0.000	0.999	0.000	0.000	0.016	0.003	0.000	0.000
obsession	0.000	0.150	0.000	0.123	0.513	0.337	0.089	0.049	0.218	0.145	0.008	0.000	0.762	0.030	0.001	0.018
audial hallucination	0.031	0.777	0.325	0.663	0.341	0.319	0.133	0.608	0.320	0.101	0.064	0.282	0.269	0.489	0.000	0.665
visual halucination	0.009	0.637	0.242	0.993	0.436	0.561	0.149	0.944	0.595	0.772	0.026	0.302	0.273	0.448	0.002	0.098
headache	0.408	0.457	0.066	0.526	0.998	0.617	0.469	0.313	0.184	0.993	0.020	0.002	0.078	0.090	0.596	0.347
subjective physical health problems	0.011	1.000	0.038	0.062	0.441	0.285	0.042	0.105	0.286	0.679	0.783	0.163	0.000	0.079	0.001	0.000
subjective mental health problems	0.000	0.596	0.000	0.004	0.442	0.406	0.374	0.137	0.222	0.557	0.113	0.357	0.023	0.547	0.877	0.329
diagnosed psychiatric disorders	0.371	0.001	0.001	0.028	0.379	0.761	0.080	0.002	0.001	0.045	0.000	0.000	0.000	0.364	0.000	0.647
non-diagnosed psychiatric disorders	0.178	0.010	0.004	0.948	0.385	0.232	0.080	0.172	0.212	0.151	0.000	0.000	0.000	0.058	0.000	0.924
psychiatric disorders total number	0.099	0.000	0.000	0.265	0.584	0.207	0.087	0.037	0.002	0.060	0.000	0.000	0.000	0.322	0.000	0.573
partner's diagnosed psychiatric disorders	0.912	0.684	0.912	0.004	0.988	0.674	0.207	0.417	0.007	0.376	0.071	0.000	0.977	0.645	0.000	0.570
partner's non-diagnosed psychiatric disord.	0.039	0.831	0.157	0.008	0.000	0.478	0.814	0.068	0.000	0.000	0.828	0.129	0.044	0.625	0.005	0.881
partner's psychiatric disord. total number	0.403	0.973	0.590	0.001	0.130	0.258	0.475	0.190	0.000	0.011	0.269	0.000	0.311	0.173	0.000	0.753
mental health problems score	0.008	0.000	0.000	0.475	0.405	0.289	0.807	0.090	0.000	0.667	0.000	0.000	0.001	0.476	0.000	0.361
physical health problems score	0.775	0.183	0.367	0.644	0.424	0.000	0.246	0.162	0.763	0.916	0.282	0.001	0.006	0.000	0.624	0.000
sexual activity	0.000	0.683	0.001	0.000	0.174	0.218	0.000	0.000	0.328	0.952	0.003	0.024	0.000	0.000	0.000	0.001
sexual desire	0.000	0.435	0.000	0.021	0.008	0.434	0.400	0.186	0.161	0.229	0.871	0.795	0.803	0.034	0.064	0.072
