# Supplementary material for: Friends with malefit. The effects of keeping dogs and cats, sustaining animal-related injuries and Toxoplasma infection on health and quality of life
Source: PLoS One. 2019 Nov 22;14(11):e0221988. doi: 10.1371/journal.pone.0221988 (PMC6874301; doi:10.1371/journal.pone.0221988)
Supplement: S10 Table — (PDF) [file pone.0221988.s025.pdf]

Table S10: Partial Kendall correlation (age, education, and urbanization controlled) between variables listed in the first raw and first column.

WOMEN WHO WERE NEVER INJURED BY A DOG

a) Partial Kendall Tau (significant Tau printed bold, no correction for multiple comparission. Blue cells and red cells indicate negative and positive correlation, respectively.)

	like dogs	like cats	refer dog	dog ever	dog now	ogs numb	dog bit	cat ever	cat now	ats numb	cat bit	: scratch	smoking	alcohol	egal dru	BMI
WHOQOL-BREF health	**0.035**	0.006	0.021	-0.002	0.015	**-0.071**	**-0.030**	**-0.047**	-0.023	**-0.040**	**-0.039**	**-0.063**	-0.017	**0.026**	-0.020	**-0.069**
WHOQOL-BREF psychological	0.014	-0.009	0.015	0.000	0.004	-0.033	-0.021	**-0.028**	-0.019	-0.028	**-0.052**	**-0.052**	**-0.042**	**-0.047**	**-0.042**	**-0.052**
WHOQOL-BREF social relationships	0.022	0.016	-0.002	-0.003	0.005	**-0.042**	-0.012	-0.015	-0.016	-0.002	**-0.034**	**-0.030**	-0.019	-0.008	-0.012	**-0.041**
WHOQOL-BREF environment	0.004	0.019	-0.011	**-0.034**	-0.012	**-0.074**	-0.016	**-0.029**	-0.004	**-0.047**	-0.023	**-0.031**	-0.012	**0.024**	**-0.029**	-0.014
WHOQOL-BREF total score	**0.024**	0.005	0.012	-0.013	0.006	**-0.064**	**-0.025**	**-0.042**	-0.019	**-0.040**	**-0.044**	**-0.054**	**-0.033**	0.006	**-0.039**	**-0.059**
children	**-0.085**	**-0.089**	0.014	**0.048**	-0.012	-0.017	**-0.035**	**0.038**	-0.016	**-0.055**	**-0.063**	**-0.062**	**-0.030**	**-0.073**	**-0.067**	**0.058**
siblings	**-0.050**	**-0.060**	0.013	**0.024**	-0.003	-0.001	**-0.031**	**0.041**	-0.001	-0.007	**-0.035**	-0.018	**-0.042**	**-0.038**	-0.012	0.010
family situation	0.011	-0.012	0.017	**-0.042**	-0.008	-0.026	-0.011	**-0.028**	**-0.026**	-0.004	**-0.040**	**-0.037**	**-0.069**	-0.013	**-0.040**	**-0.028**
economic situation	**-0.031**	**-0.033**	0.010	**-0.050**	**-0.024**	**-0.040**	-0.011	**-0.054**	**-0.035**	**-0.053**	**-0.033**	**-0.039**	**-0.080**	-0.015	**-0.047**	**-0.031**
drugs prescribed	0.006	-0.003	0.009	0.005	0.002	**0.042**	0.007	0.009	0.006	0.017	-0.004	0.019	-0.005	**-0.079**	**-0.036**	**0.114**
drugs non-prescribed	**0.025**	0.012	0.009	0.003	-0.001	0.012	0.018	0.012	0.007	-0.025	0.001	0.008	-0.015	0.001	**0.050**	-0.012
practical doctor visits	0.019	0.007	0.005	-0.005	-0.016	**0.045**	0.018	-0.006	-0.012	-0.008	0.002	**0.031**	-0.016	-0.021	**0.025**	**0.057**
antibiotics	0.014	0.008	0.005	-0.002	0.013	**0.044**	**0.027**	-0.010	0.022	-0.004	0.017	**0.036**	**0.036**	**0.028**	**0.045**	**0.040**
medical specialists visited	0.003	0.017	-0.013	0.000	-0.003	0.008	**0.039**	0.003	**0.032**	0.009	**0.044**	**0.056**	0.002	-0.013	0.010	**0.048**
anxiety	-0.022	0.016	**-0.023**	0.003	-0.017	0.014	**0.054**	0.018	**0.036**	0.013	**0.067**	**0.087**	**0.059**	**0.049**	**0.046**	-0.002
phobia	-0.017	0.020	**-0.026**	0.007	0.011	0.001	**0.039**	0.009	**0.047**	0.014	**0.029**	**0.053**	**0.043**	0.020	0.005	0.020
depression	-0.015	**0.038**	**-0.038**	0.017	-0.012	0.032	**0.040**	**0.049**	**0.057**	0.025	**0.069**	**0.073**	**0.072**	**0.066**	**0.076**	**0.034**
mania	**-0.044**	-0.021	-0.015	**0.032**	0.005	-0.019	**0.047**	0.016	0.021	0.014	**0.051**	**0.061**	**0.100**	**0.064**	**0.105**	**0.031**
obsession	**-0.047**	-0.019	-0.016	0.016	0.002	-0.025	**0.041**	**-0.025**	**0.026**	-0.004	**0.031**	**0.057**	**0.050**	**0.042**	**0.041**	0.007
audial hallucination	**-0.035**	0.000	-0.019	**0.039**	0.009	0.036	**0.031**	**0.033**	**0.041**	**0.042**	**0.036**	**0.054**	**0.059**	0.019	**0.071**	**0.036**
visual halucination	**-0.027**	-0.017	-0.006	**0.041**	0.007	0.005	**0.048**	0.024	**0.037**	0.027	**0.038**	**0.052**	**0.060**	0.009	**0.068**	**0.042**
headache	-0.009	**0.025**	-0.018	0.017	0.002	0.026	**0.038**	0.015	**0.034**	**0.040**	**0.052**	**0.069**	**0.033**	0.012	0.022	0.016
subjective physical health problems	-0.007	0.006	-0.018	-0.018	0.010	**0.060**	-0.006	0.011	0.014	0.030	0.020	**0.040**	**0.027**	**-0.060**	-0.017	**0.218**
subjective mental health problems	-0.017	0.002	-0.012	-0.014	**-0.025**	0.030	-0.009	0.011	-0.005	0.023	**0.033**	**0.049**	**0.036**	0.010	0.015	0.022
diagnosed psychiatric disorders	0.004	**0.043**	**-0.038**	**0.046**	0.013	0.013	**0.061**	**0.051**	**0.061**	0.027	**0.085**	**0.083**	**0.090**	-0.020	**0.026**	**0.057**
non-diagnosed psychiatric disorders	-0.020	**0.038**	**-0.047**	0.014	0.006	0.010	**0.041**	**0.052**	**0.046**	0.021	**0.059**	**0.043**	**0.083**	**0.041**	**0.058**	**0.034**
psychiatric disorders total number	-0.014	**0.052**	**-0.057**	**0.030**	0.014	0.009	**0.062**	**0.059**	**0.065**	0.034	**0.087**	**0.071**	**0.104**	0.020	**0.051**	**0.049**
partner's diagnosed psychiatric disorders	-0.004	0.002	-0.002	**0.050**	0.016	-0.015	0.003	**0.033**	0.014	0.021	0.002	-0.016	**0.036**	0.017	**0.052**	**0.036**
partner's non-diagnosed psychiatric disord.	-0.017	0.009	-0.017	0.018	-0.005	-0.007	-0.016	**0.037**	0.008	0.018	0.015	0.010	**0.023**	-0.021	**0.025**	**0.058**
partner's psychiatric disord. total number	-0.002	0.014	-0.011	**0.041**	0.006	-0.013	-0.003	**0.040**	0.021	0.027	0.010	-0.007	**0.034**	0.009	**0.056**	**0.039**
mental health problems score	**-0.029**	**0.038**	**-0.047**	0.018	-0.006	0.024	**0.059**	**0.036**	**0.059**	0.035	**0.085**	**0.104**	**0.084**	**0.045**	**0.064**	**0.031**
physical health problems score	**0.022**	**0.023**	0.000	0.004	-0.005	0.027	**0.025**	0.009	0.018	-0.004	**0.024**	**0.045**	0.007	-0.019	**0.026**	**0.070**
sexual activity	**0.041**	**0.057**	-0.013	**0.078**	0.015	0.037	**0.032**	**0.055**	**0.049**	0.026	**0.043**	**0.034**	**0.238**	**0.148**	**0.168**	0.011
sexual desire	**0.089**	**0.027**	**0.044**	**-0.029**	0.005	**0.060**	-0.013	**-0.034**	**-0.031**	-0.021	-0.017	**-0.031**	0.018	**0.036**	-0.001	-0.008

b) p-values of two-sided tests

	like dogs	like cats	refer dog	dog ever	dog now	ogs numb	dog bit	cat ever	cat now	ats numb	cat bit	: scratch	smoking	alcohol	egal dru	BMI
WHOQOL-BREF health	0.003	0.611	0.072	0.850	0.208	0.000	0.010	0.000	0.053	0.032	0.001	0.000	0.155	0.025	0.085	0.000
WHOQOL-BREF psychological	0.240	0.446	0.193	0.979	0.741	0.101	0.071	0.018	0.100	0.130	0.000	0.000	0.000	0.000	0.000	0.000
WHOQOL-BREF social relationships	0.067	0.178	0.860	0.807	0.646	0.036	0.310	0.202	0.169	0.922	0.004	0.010	0.106	0.506	0.321	0.000
WHOQOL-BREF environment	0.758	0.115	0.364	0.004	0.290	0.000	0.182	0.016	0.739	0.014	0.053	0.009	0.329	0.043	0.014	0.244
WHOQOL-BREF total score	0.049	0.659	0.325	0.268	0.605	0.002	0.038	0.000	0.110	0.037	0.000	0.000	0.007	0.625	0.001	0.000
children	0.000	0.000	0.192	0.000	0.262	0.349	0.001	0.000	0.144	0.001	0.000	0.000	0.007	0.000	0.000	0.000
siblings	0.000	0.000	0.236	0.025	0.802	0.975	0.004	0.000	0.950	0.678	0.001	0.096	0.000	0.001	0.276	0.325
family situation	0.317	0.248	0.110	0.000	0.454	0.150	0.317	0.009	0.013	0.807	0.000	0.000	0.000	0.247	0.000	0.008
economic situation	0.004	0.002	0.373	0.000	0.023	0.024	0.314	0.000	0.001	0.002	0.002	0.000	0.000	0.176	0.000	0.003
drugs prescribed	0.610	0.801	0.409	0.651	0.854	0.027	0.509	0.428	0.614	0.334	0.718	0.088	0.678	0.000	0.001	0.000
drugs non-prescribed	0.028	0.302	0.426	0.764	0.932	0.520	0.103	0.285	0.528	0.170	0.924	0.486	0.198	0.915	0.000	0.273
practical doctor visits	0.098	0.539	0.673	0.676	0.157	0.018	0.100	0.582	0.297	0.665	0.871	0.006	0.159	0.062	0.025	0.000
antibiotics	0.218	0.454	0.628	0.860	0.236	0.022	0.015	0.359	0.056	0.826	0.122	0.001	0.001	0.013	0.000	0.000
medical specialists visited	0.802	0.135	0.261	0.975	0.801	0.670	0.001	0.756	0.005	0.623	0.000	0.000	0.869	0.235	0.354	0.000
anxiety	0.060	0.160	0.050	0.785	0.140	0.479	0.000	0.115	0.002	0.472	0.000	0.000	0.000	0.000	0.000	0.884
phobia	0.140	0.083	0.029	0.560	0.371	0.952	0.001	0.440	0.000	0.461	0.014	0.000	0.000	0.086	0.688	0.095
depression	0.185	0.001	0.001	0.139	0.296	0.112	0.001	0.000	0.000	0.182	0.000	0.000	0.000	0.000	0.000	0.004
mania	0.000	0.082	0.206	0.009	0.677	0.362	0.000	0.181	0.084	0.477	0.000	0.000	0.000	0.000	0.000	0.012
obsession	0.000	0.121	0.198	0.189	0.895	0.220	0.001	0.042	0.034	0.818	0.010	0.000	0.000	0.001	0.001	0.570
audial hallucination	0.005	0.994	0.125	0.002	0.490	0.087	0.012	0.007	0.001	0.037	0.004	0.000	0.000	0.119	0.000	0.003
visual halucination	0.028	0.161	0.650	0.001	0.551	0.824	0.000	0.053	0.003	0.176	0.002	0.000	0.000	0.448	0.000	0.001
headache	0.430	0.033	0.131	0.134	0.891	0.183	0.001	0.188	0.003	0.032	0.000	0.000	0.004	0.292	0.063	0.166
subjective physical health problems	0.556	0.631	0.126	0.127	0.394	0.002	0.600	0.339	0.238	0.110	0.088	0.001	0.021	0.000	0.146	0.000
subjective mental health problems	0.155	0.878	0.301	0.230	0.033	0.132	0.465	0.357	0.649	0.213	0.005	0.000	0.002	0.392	0.200	0.059
diagnosed psychiatric disorders	0.744	0.000	0.001	0.000	0.266	0.510	0.000	0.000	0.000	0.141	0.000	0.000	0.000	0.077	0.022	0.000
non-diagnosed psychiatric disorders	0.082	0.001	0.000	0.228	0.599	0.589	0.000	0.000	0.000	0.243	0.000	0.000	0.000	0.000	0.000	0.003
psychiatric disorders total number	0.229	0.000	0.000	0.008	0.227	0.646	0.000	0.000	0.000	0.059	0.000	0.000	0.000	0.071	0.000	0.000
partner's diagnosed psychiatric disorders	0.718	0.892	0.872	0.000	0.159	0.431	0.795	0.004	0.213	0.246	0.830	0.146	0.001	0.124	0.000	0.002
partner's non-diagnosed psychiatric disord.	0.126	0.411	0.126	0.112	0.672	0.723	0.149	0.001	0.502	0.307	0.173	0.375	0.045	0.060	0.025	0.000
partner's psychiatric disord. total number	0.847	0.226	0.334	0.000	0.573	0.487	0.757	0.000	0.062	0.134	0.392	0.524	0.002	0.430	0.000	0.001
mental health problems score	0.009	0.001	0.000	0.104	0.592	0.211	0.000	0.001	0.000	0.051	0.000	0.000	0.000	0.000	0.000	0.005
physical health problems score	0.049	0.044	0.978	0.731	0.644	0.152	0.023	0.419	0.118	0.824	0.035	0.000	0.528	0.096	0.021	0.000
sexual activity	0.001	0.000	0.275	0.000	0.230	0.077	0.009	0.000	0.000	0.185	0.000	0.006	0.000	0.000	0.000	0.388
sexual desire	0.000	0.031	0.000	0.021	0.693	0.005	0.303	0.007	0.014	0.281	0.161	0.013	0.145	0.004	0.952	0.499
