# Supplementary material for: Friends with malefit. The effects of keeping dogs and cats, sustaining animal-related injuries and Toxoplasma infection on health and quality of life
Source: PLoS One. 2019 Nov 22;14(11):e0221988. doi: 10.1371/journal.pone.0221988 (PMC6874301; doi:10.1371/journal.pone.0221988)
Supplement: S11 Table — (PDF) [file pone.0221988.s026.pdf]

Table S11: Partial Kendall correlation (age, education, and urbanization controlled) between variables listed in the first raw and first column.

MEN WHO WERE NEVER INJURED BY A CAT

a) Partial Kendall Tau (significant Tau printed bold, no correction for multiple comparission. Blue cells and red cells indicate negative and positive correlation, respectively.)

	like dogs	like cats	refer dog	dog ever	dog now	ogs numb	dog bit	cat ever	cat now	ats numb	cat bit	scratch	smoking	alcohol	egal dru	BMI
WHOQOL-BREF health	0.027	0.013	0.004	0.006	0.014	0.033	-0.020	**0.038**	0.023	0.009	0.004	0.011	-0.012	0.009	-0.029	**-0.075**
WHOQOL-BREF psychological	**0.070**	0.000	**0.042**	**0.045**	0.029	0.050	-0.010	0.015	0.029	0.016	-0.002	-0.010	-0.011	-0.001	**-0.048**	-0.029
WHOQOL-BREF social relationships	**0.046**	-0.017	**0.036**	**0.034**	0.030	**0.068**	0.013	0.028	**0.044**	-0.009	**-0.032**	-0.022	0.015	0.007	-0.008	-0.031
WHOQOL-BREF environment	0.012	0.029	-0.019	-0.007	-0.012	0.037	-0.029	0.002	0.029	-0.028	0.011	-0.024	-0.030	0.027	-0.027	**-0.049**
WHOQOL-BREF total score	**0.050**	0.007	0.022	0.017	0.013	0.048	-0.015	0.022	0.032	-0.010	-0.006	-0.010	-0.013	0.018	**-0.034**	**-0.062**
children	**-0.059**	**-0.100**	**0.044**	**0.037**	**0.033**	**0.086**	0.004	0.014	-0.007	0.001	**-0.054**	0.002	0.026	-0.003	**-0.095**	**0.092**
siblings	**-0.030**	-0.020	-0.019	**0.041**	0.009	0.041	0.016	**0.038**	**0.033**	**0.094**	**0.032**	0.000	-0.026	**-0.039**	-0.025	-0.004
family situation	**0.030**	-0.025	**0.033**	0.004	**0.042**	0.021	-0.020	0.022	**0.042**	**-0.063**	**-0.059**	**-0.053**	-0.002	0.019	**-0.032**	-0.011
economic situation	-0.015	-0.022	0.007	**-0.047**	-0.025	-0.012	-0.021	**-0.035**	**-0.031**	-0.022	-0.028	**-0.032**	**-0.095**	0.006	**-0.072**	0.011
drugs prescribed	0.011	0.004	-0.005	0.005	-0.015	**0.094**	-0.021	**-0.056**	-0.004	**0.064**	-0.028	-0.010	-0.020	**-0.059**	**-0.069**	**0.121**
drugs non-prescribed	**0.036**	0.000	0.031	**0.054**	0.026	-0.027	-0.011	-0.008	0.002	-0.015	-0.011	0.016	**-0.055**	0.006	**0.044**	0.009
practical doctor visits	0.015	-0.017	0.019	-0.007	-0.001	**0.087**	-0.006	**-0.031**	0.003	0.015	0.002	0.019	**-0.043**	**-0.063**	**-0.048**	**0.053**
antibiotics	**-0.035**	-0.027	-0.009	-0.010	-0.002	**0.153**	-0.017	**-0.032**	0.002	0.044	-0.008	-0.003	**-0.032**	**-0.049**	-0.009	0.024
medical specialists visited	0.003	-0.029	0.017	-0.004	**0.042**	**0.070**	0.014	-0.016	-0.008	**-0.074**	0.009	0.012	**-0.045**	**-0.052**	**-0.033**	**0.033**
anxiety	-0.013	**0.046**	**-0.041**	-0.024	0.008	-0.050	**0.034**	-0.021	0.012	-0.049	0.024	0.000	0.020	0.014	**0.105**	-0.021
phobia	**-0.039**	0.028	**-0.052**	**-0.034**	0.000	-0.018	0.031	-0.031	0.006	0.006	0.027	-0.012	-0.016	0.021	**0.047**	-0.011
depression	-0.008	**0.038**	**-0.034**	0.019	**0.035**	-0.040	**0.049**	-0.003	-0.001	-0.027	0.014	-0.015	**0.048**	**0.038**	**0.114**	0.011
mania	-0.009	**0.051**	**-0.044**	0.010	0.033	-0.030	**0.083**	**0.043**	**0.060**	-0.065	**0.039**	0.021	**0.046**	**0.055**	**0.133**	-0.028
obsession	-0.031	0.022	**-0.041**	-0.016	-0.002	-0.044	0.018	-0.010	-0.022	0.026	-0.019	-0.006	-0.025	**0.044**	**0.085**	-0.012
audial hallucination	-0.022	-0.001	-0.013	0.006	0.019	-0.056	0.007	0.012	0.011	-0.051	-0.012	-0.004	0.004	0.026	**0.077**	-0.021
visual halucination	-0.025	-0.005	-0.007	0.003	0.009	-0.030	0.003	-0.011	0.000	0.021	-0.007	-0.023	0.015	**0.036**	**0.088**	**-0.041**
headache	-0.008	0.017	-0.026	**-0.036**	0.015	-0.060	0.032	-0.011	-0.008	0.041	-0.006	0.011	**-0.042**	-0.032	-0.029	0.026
subjective physical health problems	**-0.044**	0.016	**-0.048**	**-0.034**	-0.010	0.014	-0.013	**-0.032**	**-0.032**	-0.016	0.016	-0.007	**0.073**	-0.016	0.003	**0.180**
subjective mental health problems	**-0.034**	**0.038**	**-0.046**	**-0.039**	-0.020	0.007	0.007	-0.026	-0.030	0.022	0.028	0.007	0.007	-0.007	**0.035**	-0.001
diagnosed psychiatric disorders	0.028	**0.031**	-0.005	**0.051**	0.020	-0.019	**0.062**	0.024	0.021	-0.005	**0.042**	0.020	**0.057**	**-0.040**	**0.034**	**0.044**
non-diagnosed psychiatric disorders	-0.002	**0.039**	**-0.037**	0.019	0.011	-0.048	**0.035**	-0.028	-0.020	0.048	**0.043**	**0.032**	**0.068**	0.020	**0.091**	0.013
psychiatric disorders total number	0.006	**0.038**	**-0.031**	**0.032**	0.013	-0.041	**0.053**	-0.014	-0.001	0.041	**0.049**	**0.033**	**0.067**	-0.006	**0.081**	0.028
partner's diagnosed psychiatric disorders	-0.021	0.012	-0.027	0.024	0.003	-0.020	**0.037**	0.014	**0.037**	0.052	0.013	**0.042**	0.019	0.002	**0.042**	-0.003
partner's non-diagnosed psychiatric disord.	-0.013	0.019	-0.019	-0.004	0.002	-0.001	0.014	**0.056**	**0.099**	**0.126**	0.030	**0.031**	0.013	**-0.031**	0.018	-0.024
partner's psychiatric disord. total number	-0.020	0.016	-0.026	0.013	0.001	-0.028	**0.037**	**0.042**	**0.080**	**0.118**	0.016	**0.041**	0.015	-0.008	0.023	-0.013
mental health problems score	0.000	**0.048**	**-0.044**	-0.003	0.016	**-0.072**	**0.047**	-0.011	0.015	0.003	**0.035**	0.017	0.027	0.006	**0.095**	-0.005
physical health problems score	0.027	-0.015	0.023	0.022	0.020	**0.104**	-0.005	**-0.036**	0.001	-0.017	-0.008	0.011	**-0.049**	**-0.062**	-0.027	**0.063**
sexual activity	**0.064**	0.012	0.031	**0.102**	-0.018	0.030	**0.060**	**0.069**	0.031	0.009	**0.066**	**0.042**	**0.216**	**0.108**	**0.159**	**0.060**
sexual desire	**0.069**	0.004	**0.056**	**0.047**	**0.076**	0.042	0.006	0.003	-0.028	0.012	-0.028	-0.014	0.016	**0.045**	-0.006	**0.036**

b) p-values of two-sided tests

	like dogs	like cats	refer dog	dog ever	dog now	ogs numb	dog bit	cat ever	cat now	ats numb	cat bit	scratch	smoking	alcohol	egal dru	BMI
WHOQOL-BREF health	0.091	0.429	0.819	0.723	0.387	0.280	0.218	0.019	0.158	0.786	0.819	0.495	0.459	0.580	0.070	0.000
WHOQOL-BREF psychological	0.000	0.983	0.010	0.005	0.075	0.100	0.523	0.347	0.071	0.620	0.879	0.546	0.514	0.936	0.003	0.077
WHOQOL-BREF social relationships	0.004	0.283	0.028	0.035	0.061	0.026	0.427	0.086	0.006	0.775	0.046	0.173	0.346	0.667	0.604	0.054
WHOQOL-BREF environment	0.474	0.073	0.234	0.680	0.452	0.227	0.069	0.919	0.076	0.399	0.500	0.137	0.062	0.092	0.097	0.002
WHOQOL-BREF total score	0.002	0.677	0.185	0.292	0.427	0.125	0.366	0.173	0.052	0.754	0.713	0.523	0.427	0.263	0.041	0.000
children	0.000	0.000	0.003	0.012	0.022	0.001	0.781	0.327	0.614	0.962	0.000	0.881	0.090	0.833	0.000	0.000
siblings	0.041	0.182	0.200	0.005	0.557	0.127	0.261	0.009	0.024	0.001	0.028	0.989	0.090	0.013	0.103	0.798
family situation	0.040	0.091	0.024	0.788	0.005	0.427	0.162	0.135	0.005	0.030	0.000	0.000	0.897	0.211	0.038	0.444
economic situation	0.309	0.136	0.650	0.001	0.091	0.664	0.142	0.018	0.034	0.445	0.058	0.030	0.000	0.696	0.000	0.452
drugs prescribed	0.496	0.812	0.751	0.765	0.343	0.001	0.173	0.000	0.784	0.040	0.067	0.508	0.206	0.000	0.000	0.000
drugs non-prescribed	0.020	0.990	0.052	0.000	0.099	0.344	0.498	0.614	0.922	0.644	0.485	0.308	0.000	0.708	0.005	0.580
practical doctor visits	0.324	0.275	0.228	0.634	0.950	0.003	0.697	0.043	0.853	0.629	0.912	0.222	0.005	0.000	0.002	0.001
antibiotics	0.026	0.089	0.561	0.513	0.893	0.000	0.274	0.039	0.882	0.159	0.598	0.837	0.039	0.002	0.574	0.128
medical specialists visited	0.853	0.066	0.284	0.816	0.007	0.016	0.367	0.303	0.613	0.019	0.562	0.451	0.004	0.001	0.031	0.034
anxiety	0.407	0.005	0.013	0.138	0.642	0.098	0.035	0.189	0.455	0.143	0.141	0.991	0.213	0.394	0.000	0.195
phobia	0.019	0.094	0.002	0.043	0.986	0.568	0.060	0.066	0.719	0.871	0.103	0.477	0.329	0.214	0.004	0.508
depression	0.625	0.020	0.042	0.242	0.034	0.194	0.003	0.868	0.937	0.426	0.405	0.371	0.003	0.020	0.000	0.490
mania	0.612	0.003	0.011	0.570	0.054	0.343	0.000	0.011	0.000	0.057	0.023	0.219	0.007	0.001	0.000	0.099
obsession	0.072	0.185	0.016	0.357	0.912	0.168	0.275	0.556	0.193	0.452	0.256	0.724	0.145	0.010	0.000	0.473
audial hallucination	0.201	0.947	0.465	0.730	0.269	0.087	0.688	0.497	0.526	0.154	0.476	0.801	0.828	0.139	0.000	0.235
visual halucination	0.165	0.759	0.680	0.848	0.626	0.367	0.864	0.542	0.996	0.556	0.689	0.194	0.386	0.041	0.000	0.020
headache	0.642	0.307	0.116	0.027	0.344	0.052	0.052	0.495	0.627	0.211	0.708	0.500	0.010	0.051	0.071	0.107
subjective physical health problems	0.006	0.328	0.003	0.036	0.529	0.644	0.402	0.049	0.048	0.635	0.330	0.643	0.000	0.316	0.865	0.000
subjective mental health problems	0.035	0.020	0.005	0.016	0.213	0.813	0.654	0.103	0.066	0.492	0.082	0.652	0.675	0.669	0.029	0.966
diagnosed psychiatric disorders	0.068	0.046	0.727	0.001	0.201	0.509	0.000	0.123	0.170	0.874	0.007	0.198	0.000	0.010	0.030	0.005
non-diagnosed psychiatric disorders	0.894	0.013	0.020	0.225	0.480	0.100	0.024	0.074	0.208	0.130	0.005	0.038	0.000	0.200	0.000	0.388
psychiatric disorders total number	0.701	0.015	0.045	0.038	0.389	0.161	0.001	0.384	0.957	0.191	0.001	0.036	0.000	0.696	0.000	0.072
partner's diagnosed psychiatric disorders	0.171	0.430	0.089	0.115	0.853	0.481	0.018	0.352	0.018	0.098	0.388	0.006	0.232	0.897	0.007	0.845
partner's non-diagnosed psychiatric disord.	0.404	0.220	0.236	0.804	0.888	0.970	0.379	0.000	0.000	0.000	0.052	0.043	0.415	0.043	0.254	0.121
partner's psychiatric disord. total number	0.193	0.311	0.094	0.406	0.949	0.326	0.017	0.007	0.000	0.000	0.312	0.007	0.320	0.623	0.146	0.393
mental health problems score	0.996	0.002	0.005	0.832	0.297	0.013	0.003	0.476	0.332	0.915	0.025	0.282	0.077	0.684	0.000	0.745
physical health problems score	0.079	0.333	0.137	0.159	0.204	0.000	0.770	0.021	0.947	0.588	0.624	0.462	0.002	0.000	0.077	0.000
sexual activity	0.000	0.455	0.061	0.000	0.270	0.336	0.000	0.000	0.060	0.775	0.000	0.011	0.000	0.000	0.000	0.000
sexual desire	0.000	0.800	0.001	0.004	0.000	0.175	0.736	0.843	0.088	0.716	0.086	0.407	0.340	0.006	0.716	0.027
