# Supplementary material for: Friends with malefit. The effects of keeping dogs and cats, sustaining animal-related injuries and Toxoplasma infection on health and quality of life
Source: PLoS One. 2019 Nov 22;14(11):e0221988. doi: 10.1371/journal.pone.0221988 (PMC6874301; doi:10.1371/journal.pone.0221988)
Supplement: S12 Table — (PDF) [file pone.0221988.s027.pdf]

Table S12: Partial Kendall correlation (age, education, and urbanization controlled) between variables listed in the first raw and first column.

WOMEN WHO WERE NEVER INJURED BY A CAT

a) Partial Kendall Tau (significant Tau printed bold, no correction for multiple comparission. Blue cells and red cells indicate negative and positive correlation, respectively.)

	like dogs	like cats	refer dog	dog ever	dog now	ogs numb	dog bit	cat ever	cat now	ats numb	cat bit	scratch	smoking	alcohol	legal dru	BMI
WHOQOL-BREF health	**0.028**	0.017	0.004	-0.010	0.013	-0.033	-0.017	**-0.036**	-0.012	0.002	**-0.042**	**-0.034**	-0.004	0.021	**-0.031**	**-0.085**
WHOQOL-BREF psychological	0.013	0.012	-0.009	0.010	0.004	-0.015	-0.013	-0.002	0.010	0.018	**-0.032**	**-0.036**	**-0.051**	**-0.031**	**-0.049**	**-0.055**
WHOQOL-BREF social relationships	**0.031**	**0.035**	-0.010	-0.004	0.003	0.010	-0.013	0.002	-0.009	0.023	-0.016	-0.014	-0.016	-0.004	-0.020	**-0.043**
WHOQOL-BREF environment	-0.003	0.021	-0.024	**-0.048**	-0.007	-0.031	**-0.035**	-0.013	-0.005	-0.022	-0.020	-0.004	0.001	**0.044**	-0.019	**-0.039**
WHOQOL-BREF total score	0.019	0.020	-0.009	-0.018	0.004	-0.015	-0.023	-0.022	-0.008	0.000	**-0.040**	**-0.028**	**-0.029**	0.012	**-0.038**	**-0.073**
children	**-0.100**	**-0.070**	-0.004	0.017	**-0.024**	-0.029	-0.006	**0.057**	0.000	-0.040	**-0.039**	**-0.027**	**-0.049**	**-0.067**	**-0.071**	**0.071**
siblings	**-0.072**	**-0.049**	-0.010	0.010	**-0.033**	0.008	-0.011	**0.066**	0.022	0.004	-0.022	-0.015	-0.024	-0.023	0.006	0.010
family situation	0.018	0.003	-0.002	-0.021	-0.009	-0.022	-0.023	-0.002	-0.011	-0.010	**-0.028**	**-0.035**	**-0.067**	-0.012	-0.023	**-0.043**
economic situation	**-0.043**	**-0.038**	0.000	**-0.037**	**-0.024**	-0.034	**-0.032**	**-0.045**	**-0.034**	**-0.046**	**-0.040**	**-0.027**	**-0.090**	**-0.027**	**-0.045**	**-0.037**
drugs prescribed	-0.004	-0.010	0.011	-0.004	-0.003	0.001	-0.009	-0.001	-0.016	0.009	**-0.031**	-0.001	**-0.036**	**-0.068**	**-0.028**	**0.119**
drugs non-prescribed	**0.041**	0.009	0.022	**0.030**	-0.011	-0.023	**0.040**	0.009	0.006	0.011	-0.003	-0.002	**-0.027**	**-0.027**	**0.061**	-0.001
practical doctor visits	-0.001	0.002	0.000	-0.019	**-0.030**	-0.021	0.008	0.011	-0.014	0.012	0.014	0.018	-0.019	-0.014	0.024	**0.069**
antibiotics	-0.002	0.009	-0.007	-0.002	**-0.029**	0.013	0.013	-0.020	0.000	0.005	**0.026**	0.020	**0.027**	0.005	0.013	**0.044**
medical specialists visited	0.001	-0.004	0.006	-0.025	**-0.030**	**-0.056**	**0.026**	-0.005	0.011	-0.020	0.024	0.024	-0.009	-0.022	0.007	**0.050**
anxiety	-0.011	0.005	-0.003	0.005	-0.015	0.040	**0.055**	0.001	0.023	-0.004	**0.058**	**0.056**	**0.057**	**0.061**	**0.043**	0.001
phobia	-0.024	0.024	**-0.031**	0.006	0.008	0.032	**0.053**	0.000	**0.048**	0.010	**0.055**	**0.044**	**0.038**	**0.032**	0.017	0.022
depression	-0.011	**0.026**	-0.022	0.023	-0.016	**0.064**	**0.054**	**0.050**	**0.053**	0.005	**0.067**	**0.050**	**0.080**	**0.065**	**0.072**	**0.046**
mania	**-0.059**	-0.026	-0.008	**0.033**	-0.016	0.010	**0.036**	0.016	0.016	-0.010	**0.055**	**0.058**	**0.087**	**0.093**	**0.097**	**0.031**
obsession	**-0.055**	-0.022	-0.012	0.020	-0.011	0.008	**0.049**	-0.005	**0.027**	0.007	**0.042**	**0.032**	**0.049**	**0.069**	**0.069**	0.003
audial hallucination	**-0.028**	-0.012	0.003	**0.043**	0.002	**0.049**	**0.048**	0.019	**0.033**	0.019	**0.036**	**0.041**	**0.066**	**0.053**	**0.095**	**0.028**
visual halucination	-0.017	-0.026	0.017	**0.059**	0.018	0.025	**0.065**	0.024	**0.030**	0.023	**0.028**	**0.029**	**0.067**	0.026	**0.076**	**0.048**
headache	-0.019	0.015	-0.018	0.008	0.001	**0.042**	**0.038**	0.001	**0.041**	0.027	**0.063**	**0.027**	**0.031**	0.012	**0.026**	0.000
subjective physical health problems	-0.011	0.023	**-0.033**	-0.025	-0.006	0.035	-0.025	-0.001	**0.028**	-0.005	0.003	-0.012	**0.051**	**-0.047**	-0.019	**0.224**
subjective mental health problems	0.005	-0.016	0.019	-0.004	-0.002	0.009	0.006	-0.020	**-0.030**	-0.038	0.004	0.001	**0.032**	0.009	0.020	0.012
diagnosed psychiatric disorders	**0.035**	**0.042**	-0.016	**0.039**	0.003	-0.003	**0.070**	**0.027**	**0.040**	-0.009	**0.083**	**0.045**	**0.100**	-0.023	**0.041**	**0.046**
non-diagnosed psychiatric disorders	-0.020	**0.040**	**-0.044**	-0.004	-0.001	**0.053**	**0.055**	**0.041**	**0.038**	0.036	**0.097**	**0.067**	**0.077**	**0.046**	**0.085**	**0.026**
psychiatric disorders total number	0.000	**0.050**	**-0.044**	0.012	-0.003	0.024	**0.080**	**0.037**	**0.046**	0.024	**0.109**	**0.066**	**0.105**	0.024	**0.078**	**0.042**
partner's diagnosed psychiatric disorders	-0.007	0.002	0.000	**0.025**	-0.001	-0.011	0.006	**0.028**	-0.008	0.035	**0.032**	**-0.054**	**0.039**	0.007	**0.073**	0.020
partner's non-diagnosed psychiatric disord.	0.010	**0.026**	-0.005	0.010	0.008	-0.007	-0.018	**0.058**	-0.006	0.027	**0.045**	-0.004	-0.003	**-0.026**	**0.026**	**0.052**
partner's psychiatric disord. total number	0.008	0.023	-0.005	**0.030**	0.003	0.003	0.001	**0.053**	-0.003	0.041	**0.047**	**-0.036**	**0.026**	0.003	**0.065**	**0.032**
mental health problems score	-0.021	0.025	**-0.030**	0.010	-0.007	**0.042**	**0.075**	0.022	**0.046**	0.008	**0.094**	**0.059**	**0.088**	**0.053**	**0.069**	**0.027**
physical health problems score	0.014	0.005	0.009	-0.002	**-0.029**	**-0.041**	0.024	0.004	-0.006	0.006	0.014	0.021	-0.011	**-0.033**	0.024	**0.081**
sexual activity	**0.045**	**0.052**	-0.005	**0.055**	0.014	0.037	**0.046**	**0.050**	**0.043**	0.000	**0.070**	0.026	**0.253**	**0.142**	**0.160**	0.016
sexual desire	**0.105**	0.041	0.029	0.013	**0.034**	**0.049**	**0.039**	-0.025	**-0.032**	-0.016	0.002	-0.023	**0.043**	0.020	-0.001	-0.007

b) p-values of two-sided tests

	like dogs	like cats	refer dog	dog ever	dog now	ogs numb	dog bit	cat ever	cat now	ats numb	cat bit	scratch	smoking	alcohol	legal dru	BMI
WHOQOL-BREF health	0.033	0.210	0.781	0.444	0.319	0.127	0.196	0.007	0.385	0.921	0.002	0.011	0.791	0.113	0.020	0.000
WHOQOL-BREF psychological	0.345	0.350	0.512	0.467	0.745	0.497	0.320	0.892	0.459	0.451	0.015	0.007	0.000	0.019	0.000	0.000
WHOQOL-BREF social relationships	0.019	0.008	0.476	0.743	0.836	0.650	0.325	0.882	0.498	0.316	0.223	0.275	0.239	0.752	0.132	0.001
WHOQOL-BREF environment	0.800	0.121	0.074	0.000	0.625	0.145	0.007	0.323	0.718	0.339	0.129	0.761	0.960	0.001	0.161	0.004
WHOQOL-BREF total score	0.155	0.149	0.531	0.180	0.764	0.501	0.091	0.097	0.530	0.995	0.003	0.038	0.034	0.358	0.005	0.000
children	0.000	0.000	0.710	0.151	0.044	0.134	0.638	0.000	0.976	0.056	0.001	0.024	0.000	0.000	0.000	0.000
siblings	0.000	0.000	0.413	0.383	0.006	0.689	0.360	0.000	0.065	0.844	0.064	0.201	0.057	0.066	0.662	0.385
family situation	0.143	0.771	0.895	0.073	0.472	0.266	0.053	0.839	0.361	0.638	0.019	0.003	0.000	0.352	0.065	0.000
economic situation	0.000	0.002	0.977	0.002	0.045	0.075	0.008	0.000	0.004	0.029	0.001	0.021	0.000	0.031	0.000	0.002
drugs prescribed	0.757	0.436	0.387	0.779	0.796	0.956	0.486	0.941	0.218	0.704	0.015	0.950	0.004	0.000	0.028	0.000
drugs non-prescribed	0.001	0.475	0.084	0.016	0.397	0.262	0.001	0.491	0.635	0.627	0.805	0.863	0.035	0.031	0.000	0.941
practical doctor visits	0.931	0.868	0.980	0.134	0.020	0.305	0.537	0.398	0.276	0.579	0.261	0.157	0.127	0.255	0.055	0.000
antibiotics	0.852	0.480	0.570	0.901	0.023	0.542	0.296	0.109	0.995	0.816	0.037	0.113	0.031	0.701	0.324	0.001
medical specialists visited	0.947	0.726	0.646	0.052	0.017	0.007	0.041	0.674	0.365	0.381	0.055	0.062	0.500	0.088	0.594	0.000
anxiety	0.416	0.702	0.848	0.692	0.262	0.058	0.000	0.931	0.072	0.855	0.000	0.000	0.000	0.000	0.001	0.938
phobia	0.078	0.070	0.022	0.672	0.541	0.142	0.000	0.995	0.000	0.679	0.000	0.001	0.004	0.018	0.193	0.105
depression	0.413	0.048	0.100	0.084	0.236	0.003	0.000	0.000	0.000	0.840	0.000	0.000	0.000	0.000	0.000	0.000
mania	0.000	0.056	0.583	0.017	0.244	0.641	0.009	0.244	0.231	0.682	0.000	0.000	0.000	0.000	0.000	0.023
obsession	0.000	0.102	0.384	0.138	0.418	0.716	0.000	0.705	0.047	0.764	0.002	0.019	0.000	0.000	0.000	0.846
audial hallucination	0.049	0.376	0.835	0.002	0.897	0.032	0.001	0.175	0.019	0.445	0.011	0.004	0.000	0.000	0.000	0.049
visual halucination	0.231	0.062	0.242	0.000	0.199	0.285	0.000	0.084	0.035	0.357	0.046	0.038	0.000	0.060	0.000	0.001
headache	0.150	0.264	0.184	0.523	0.962	0.049	0.004	0.927	0.002	0.255	0.000	0.041	0.019	0.373	0.049	0.972
subjective physical health problems	0.408	0.086	0.013	0.061	0.628	0.105	0.053	0.952	0.035	0.833	0.837	0.382	0.000	0.000	0.154	0.000
subjective mental health problems	0.681	0.233	0.161	0.736	0.895	0.691	0.643	0.129	0.025	0.105	0.749	0.917	0.017	0.499	0.133	0.372
diagnosed psychiatric disorders	0.006	0.001	0.210	0.002	0.797	0.890	0.000	0.033	0.002	0.690	0.000	0.000	0.000	0.068	0.001	0.000
non-diagnosed psychiatric disorders	0.108	0.002	0.001	0.772	0.921	0.010	0.000	0.001	0.003	0.109	0.000	0.000	0.000	0.000	0.000	0.042
psychiatric disorders total number	0.971	0.000	0.001	0.363	0.830	0.251	0.000	0.004	0.000	0.289	0.000	0.000	0.000	0.060	0.000	0.001
partner's diagnosed psychiatric disorders	0.556	0.865	0.985	0.047	0.933	0.598	0.643	0.026	0.548	0.118	0.013	0.000	0.002	0.592	0.000	0.125
partner's non-diagnosed psychiatric disord.	0.426	0.039	0.669	0.422	0.529	0.721	0.147	0.000	0.649	0.226	0.000	0.775	0.822	0.041	0.038	0.000
partner's psychiatric disord. total number	0.510	0.073	0.692	0.018	0.819	0.891	0.947	0.000	0.795	0.069	0.000	0.005	0.038	0.832	0.000	0.011
mental health problems score	0.094	0.050	0.018	0.419	0.556	0.040	0.000	0.084	0.000	0.734	0.000	0.000	0.000	0.000	0.000	0.032
physical health problems score	0.256	0.697	0.469	0.874	0.022	0.048	0.063	0.745	0.620	0.793	0.257	0.097	0.387	0.008	0.060	0.000
sexual activity	0.001	0.000	0.710	0.000	0.303	0.097	0.001	0.000	0.002	0.985	0.000	0.061	0.000	0.000	0.000	0.254
sexual desire	0.000	0.004	0.038	0.347	0.017	0.031	0.005	0.071	0.023	0.521	0.876	0.106	0.002	0.148	0.962	0.614
