# Supplementary material for: Friends with malefit. The effects of keeping dogs and cats, sustaining animal-related injuries and Toxoplasma infection on health and quality of life
Source: PLoS One. 2019 Nov 22;14(11):e0221988. doi: 10.1371/journal.pone.0221988 (PMC6874301; doi:10.1371/journal.pone.0221988)
Supplement: S13 Table — (PDF) [file pone.0221988.s028.pdf]

Table S13: Partial Kendall correlation (age, education, and urbanization controlled) between variables listed in the first raw and first column.

TOXOPLASMA-FREE MEN

a) Partial Kendall Tau (significant Tau printed bold, no correction for multiple comparission. Blue cells and red cells indicate negative and positive correlation, respectively.)

	like dogs	like cats	prefer dogs	dog ever	dog now	dogs number	dog bit	cat ever	cat now	cats number	cat bit	cat scratched	smoking	alcohol	illegal drugs	BMI
WHOQOL-BREF health	0.067	0.015	0.038	**-0.097**	-0.051	-0.101	**0.094**	0.012	-0.002	0.091	-0.033	-0.005	-0.044	-0.065	-0.009	-0.049
WHOQOL-BREF psychological	**0.114**	0.011	0.064	-0.016	0.011	**-0.243**	0.060	-0.023	-0.001	0.049	-0.064	-0.076	-0.005	-0.041	-0.061	-0.045
WHOQOL-BREF social relationships	0.081	0.018	0.017	-0.005	0.059	0.019	-0.026	-0.082	0.054	0.041	-0.074	**-0.103**	0.009	-0.026	0.002	-0.064
WHOQOL-BREF environment	0.015	0.054	-0.054	-0.016	**0.133**	0.037	0.050	0.021	**0.161**	0.086	0.010	-0.017	-0.055	0.062	0.002	**-0.103**
WHOQOL-BREF total score	**0.092**	0.032	0.030	-0.061	0.023	**-0.178**	0.064	-0.029	0.050	0.075	-0.030	-0.041	-0.026	-0.022	-0.028	-0.078
children	-0.007	**-0.115**	**0.095**	0.031	0.011	-0.058	-0.040	-0.038	0.046	0.029	0.016	-0.016	0.079	0.018	-0.050	**0.110**
siblings	-0.031	-0.009	-0.036	**0.110**	0.012	-0.007	**-0.089**	**-0.092**	-0.019	-0.064	0.061	-0.040	**-0.154**	**-0.085**	0.008	-0.051
family situation	0.051	-0.048	0.075	0.062	**0.098**	0.023	**0.086**	**-0.078**	**0.080**	-0.003	-0.041	-0.051	0.063	0.066	0.044	-0.051
economic situation	**0.081**	-0.039	**0.086**	0.013	0.021	-0.037	0.025	-0.044	-0.036	-0.005	0.008	-0.021	-0.040	-0.025	-0.064	0.006
drugs prescribed	-0.040	-0.066	0.028	0.077	**0.101**	**0.334**	**-0.104**	-0.032	0.054	0.093	-0.008	-0.011	-0.036	-0.064	**-0.104**	**0.096**
drugs non-prescribed	**0.098**	**0.094**	0.008	0.045	**0.085**	-0.059	0.046	-0.021	0.043	-0.005	0.016	-0.009	-0.021	0.029	**0.117**	0.032
practical doctor visits	0.006	0.004	0.001	0.003	0.023	0.119	-0.031	0.031	**0.093**	**0.199**	-0.003	-0.036	**-0.121**	-0.066	0.010	0.036
antibiotics	-0.023	-0.006	-0.022	-0.056	0.048	0.130	0.015	0.073	**0.109**	-0.081	0.041	0.065	**-0.085**	0.005	0.000	0.036
medical specialists visited	0.037	-0.026	0.048	**0.117**	**0.091**	0.125	**0.113**	**0.098**	**0.089**	-0.098	**0.123**	0.051	0.005	0.034	0.032	**0.092**
anxiety	**-0.098**	0.071	**-0.103**	0.039	0.041	0.130	0.008	**0.096**	**0.101**	-0.122	**0.127**	0.073	**0.087**	0.033	**0.167**	-0.002
phobia	**-0.091**	**0.100**	**-0.118**	0.045	0.005	0.178	-0.029	**0.104**	**0.231**	0.060	**0.163**	**0.107**	0.049	0.023	**0.158**	-0.027
depression	-0.025	0.065	-0.058	**0.134**	**0.130**	**0.248**	0.013	**0.136**	**0.119**	-0.035	**0.109**	0.056	**0.110**	0.028	**0.125**	0.054
mania	-0.057	0.028	-0.061	-0.067	-0.036	**0.206**	-0.045	0.000	0.070	-0.068	0.068	-0.027	**0.124**	**0.117**	**0.217**	-0.075
obsession	-0.050	0.076	-0.085	0.067	**0.106**	0.147	-0.020	**0.152**	**0.180**	0.099	0.042	-0.002	0.064	0.065	**0.106**	-0.029
audial hallucination	0.005	0.008	0.006	0.033	0.076	0.153	0.008	**0.104**	0.048	-0.131	**0.098**	0.016	**0.148**	**0.101**	**0.237**	0.018
visual halucination	-0.033	0.013	-0.031	0.019	0.060	0.109	0.007	0.079	**0.141**	-0.019	0.050	-0.024	**0.180**	**0.150**	**0.220**	-0.032
headache	-0.053	0.060	**-0.116**	0.054	**0.089**	0.059	-0.049	**0.121**	**0.087**	0.023	0.049	0.003	-0.016	-0.018	0.037	0.003
subjective physical health problems	**-0.132**	0.014	**-0.094**	0.041	-0.039	0.081	-0.015	0.063	0.074	0.063	**0.095**	0.047	0.067	0.006	-0.003	**0.091**
subjective mental health problems	**-0.129**	0.017	**-0.090**	0.081	0.041	**0.292**	0.001	0.058	0.076	-0.013	0.085	**0.106**	0.040	-0.016	**0.090**	0.000
diagnosed psychiatric disorders	0.082	0.058	0.046	0.058	0.077	**0.174**	**0.129**	**0.111**	**0.107**	0.105	**0.128**	0.040	**0.094**	**-0.084**	**0.091**	0.029
non-diagnosed psychiatric disorders	-0.041	**0.112**	**-0.103**	0.042	**-0.101**	-0.114	0.042	0.047	**0.085**	-0.017	**0.150**	**0.124**	-0.009	0.033	**0.134**	0.073
psychiatric disorders total number	0.010	**0.104**	-0.048	0.075	-0.023	0.074	**0.083**	**0.100**	**0.147**	0.083	**0.188**	**0.119**	0.078	-0.026	**0.111**	0.075
partner's diagnosed psychiatric disorders	**0.099**	**0.085**	0.019	**0.138**	**0.117**	0.080	**0.087**	0.041	0.064	0.113	0.014	0.077	-0.031	0.007	0.019	-0.049
partner's non-diagnosed psychiatric disord.	**0.087**	0.071	0.012	-0.058	0.025	0.104	-0.041	0.073	**0.180**	0.046	-0.072	-0.018	-0.078	-0.061	-0.015	-0.073
partner's psychiatric disord. total number	**0.107**	**0.095**	0.009	-0.003	0.052	0.024	0.020	0.079	**0.190**	0.064	-0.014	0.058	-0.073	-0.009	0.028	**-0.084**
mental health problems score	-0.038	**0.102**	**-0.089**	**0.096**	**0.090**	**0.163**	0.050	**0.171**	**0.160**	0.068	**0.138**	**0.102**	0.081	0.020	**0.134**	0.048
physical health problems score	0.053	0.004	0.035	0.060	0.081	**0.209**	-0.007	0.033	**0.101**	0.045	0.066	0.053	-0.052	-0.031	0.054	**0.099**
sexual activity	0.074	**-0.091**	**0.104**	0.003	-0.010	-0.014	0.039	-0.049	-0.009	-0.114	-0.042	-0.011	**0.197**	**0.216**	**0.122**	0.052
sexual desire	**0.103**	0.020	0.058	0.015	0.005	**-0.390**	0.035	0.006	-0.044	-0.094	0.002	0.066	-0.044	0.025	-0.026	0.018

b) p-values of two-sided tests

	like dogs	like cats	prefer dogs	dog ever	dog now	dogs number	dog bit	cat ever	cat now	cats number	cat bit	cat scratched	smoking	alcohol	illegal drugs	BMI
WHOQOL-BREF health	0.130	0.725	0.392	0.025	0.236	0.239	0.031	0.782	0.954	0.254	0.452	0.904	0.310	0.137	0.834	0.260
WHOQOL-BREF psychological	0.010	0.797	0.145	0.714	0.805	0.004	0.171	0.591	0.985	0.538	0.145	0.083	0.903	0.350	0.162	0.297
WHOQOL-BREF social relationships	0.067	0.686	0.707	0.910	0.177	0.828	0.550	0.060	0.214	0.606	0.092	0.019	0.833	0.556	0.966	0.143
WHOQOL-BREF environment	0.739	0.217	0.221	0.707	0.002	0.669	0.247	0.622	0.000	0.276	0.826	0.695	0.206	0.151	0.964	0.018
WHOQOL-BREF total score	0.045	0.478	0.515	0.174	0.602	0.046	0.153	0.525	0.269	0.366	0.513	0.365	0.560	0.620	0.537	0.083
children	0.847	0.003	0.015	0.418	0.773	0.422	0.301	0.322	0.229	0.665	0.683	0.679	0.057	0.658	0.225	0.002
siblings	0.425	0.813	0.355	0.004	0.754	0.918	0.021	0.017	0.628	0.340	0.114	0.305	0.000	0.040	0.847	0.161
family situation	0.192	0.210	0.054	0.107	0.010	0.745	0.025	0.042	0.038	0.967	0.282	0.191	0.132	0.113	0.292	0.157
economic situation	0.037	0.314	0.028	0.734	0.588	0.609	0.518	0.246	0.347	0.941	0.842	0.583	0.331	0.539	0.121	0.870
drugs prescribed	0.348	0.113	0.509	0.067	0.016	0.000	0.013	0.450	0.196	0.216	0.850	0.795	0.390	0.127	0.013	0.021
drugs non-prescribed	0.021	0.024	0.856	0.282	0.043	0.473	0.273	0.615	0.305	0.952	0.698	0.833	0.613	0.480	0.005	0.437
practical doctor visits	0.880	0.932	0.973	0.937	0.576	0.149	0.457	0.453	0.026	0.008	0.948	0.388	0.004	0.116	0.807	0.382
antibiotics	0.590	0.887	0.611	0.180	0.248	0.113	0.724	0.080	0.009	0.281	0.333	0.123	0.042	0.907	0.997	0.394
medical specialists visited	0.377	0.527	0.259	0.005	0.028	0.126	0.007	0.019	0.032	0.189	0.004	0.228	0.904	0.421	0.443	0.027
anxiety	0.026	0.105	0.019	0.364	0.343	0.136	0.856	0.028	0.021	0.127	0.004	0.094	0.046	0.448	0.000	0.966
phobia	0.044	0.025	0.009	0.307	0.914	0.050	0.515	0.020	0.000	0.446	0.000	0.017	0.273	0.611	0.000	0.546
depression	0.568	0.136	0.185	0.002	0.003	0.004	0.769	0.002	0.006	0.656	0.013	0.200	0.011	0.525	0.004	0.217
mania	0.215	0.543	0.185	0.143	0.438	0.025	0.333	0.995	0.127	0.418	0.141	0.553	0.007	0.011	0.000	0.105
obsession	0.267	0.092	0.060	0.132	0.018	0.104	0.652	0.001	0.000	0.230	0.352	0.962	0.151	0.143	0.018	0.516
audial hallucination	0.910	0.867	0.897	0.470	0.100	0.103	0.867	0.024	0.297	0.125	0.035	0.724	0.001	0.029	0.000	0.690
visual halucination	0.483	0.780	0.507	0.678	0.197	0.253	0.876	0.091	0.002	0.828	0.282	0.610	0.000	0.001	0.000	0.498
headache	0.228	0.169	0.009	0.215	0.040	0.499	0.263	0.005	0.044	0.775	0.259	0.946	0.710	0.680	0.397	0.943
subjective physical health problems	0.003	0.740	0.032	0.348	0.364	0.350	0.732	0.143	0.086	0.426	0.030	0.284	0.122	0.895	0.939	0.035
subjective mental health problems	0.003	0.691	0.040	0.062	0.343	0.001	0.989	0.183	0.077	0.872	0.050	0.015	0.357	0.710	0.038	0.994
diagnosed psychiatric disorders	0.054	0.167	0.282	0.168	0.067	0.035	0.002	0.008	0.010	0.161	0.002	0.340	0.025	0.044	0.030	0.491
non-diagnosed psychiatric disorders	0.334	0.008	0.016	0.314	0.016	0.169	0.316	0.263	0.043	0.818	0.000	0.003	0.824	0.426	0.001	0.079
psychiatric disorders total number	0.813	0.014	0.257	0.072	0.582	0.375	0.047	0.017	0.000	0.271	0.000	0.005	0.063	0.541	0.008	0.072
partner's diagnosed psychiatric disorders	0.019	0.044	0.650	0.001	0.005	0.337	0.037	0.332	0.128	0.134	0.743	0.068	0.464	0.863	0.656	0.238
partner's non-diagnosed psychiatric disord.	0.040	0.089	0.781	0.168	0.553	0.207	0.332	0.081	0.000	0.542	0.087	0.678	0.061	0.145	0.713	0.080
partner's psychiatric disord. total number	0.012	0.023	0.826	0.944	0.218	0.773	0.641	0.058	0.000	0.401	0.743	0.173	0.084	0.833	0.501	0.044
mental health problems score	0.368	0.014	0.035	0.021	0.031	0.046	0.226	0.000	0.000	0.357	0.001	0.015	0.051	0.625	0.001	0.247
physical health problems score	0.209	0.915	0.407	0.153	0.053	0.010	0.873	0.426	0.015	0.549	0.117	0.209	0.216	0.453	0.196	0.017
sexual activity	0.098	0.040	0.020	0.951	0.817	0.875	0.371	0.263	0.831	0.152	0.346	0.801	0.000	0.000	0.006	0.234
sexual desire	0.022	0.654	0.193	0.741	0.906	0.000	0.423	0.900	0.318	0.240	0.956	0.139	0.322	0.569	0.552	0.691
