# Supplementary material for: Friends with malefit. The effects of keeping dogs and cats, sustaining animal-related injuries and Toxoplasma infection on health and quality of life
Source: PLoS One. 2019 Nov 22;14(11):e0221988. doi: 10.1371/journal.pone.0221988 (PMC6874301; doi:10.1371/journal.pone.0221988)
Supplement: S14 Table — (PDF) [file pone.0221988.s029.pdf]

Table S14: Partial Kendall correlation (age, education, and urbanization controlled) between variables listed in the first raw and first column.

TOXOPLASMA-FREE WOMEN

a) Partial Kendall Tau (significant Tau printed bold, no correction for multiple comparission. Blue cells and red cells indicate negative and positive correlation, respectively.)

	like dogs	like cats	prefer dogs	dog ever	dog now	dogs number	dog bit	cat ever	cat now	cats number	cat bit	cat scratched	smoking	alcohol	illegal drugs	BMI
WHOQOL-BREF health	0.021	0.005	0.018	-0.025	0.026	**-0.090**	-0.043	**-0.061**	-0.027	-0.022	-0.036	**-0.061**	-0.014	0.044	0.022	**-0.082**
WHOQOL-BREF psychological	-0.019	-0.015	0.005	-0.004	0.028	**-0.078**	-0.041	-0.015	-0.026	0.001	**-0.046**	-0.034	-0.040	-0.039	-0.012	**-0.061**
WHOQOL-BREF social relationships	0.027	0.041	-0.016	-0.004	0.018	-0.039	-0.036	0.015	-0.001	-0.011	-0.002	0.020	-0.004	**0.051**	-0.026	-0.041
WHOQOL-BREF environment	0.007	-0.002	0.021	-0.013	0.011	**-0.081**	-0.036	**-0.054**	**-0.086**	-0.035	-0.026	-0.034	0.033	0.040	-0.013	-0.028
WHOQOL-BREF total score	0.009	-0.002	0.017	-0.025	0.023	**-0.098**	-0.044	**-0.052**	**-0.051**	-0.032	-0.038	**-0.048**	-0.023	0.028	-0.010	**-0.080**
children	**-0.113**	**-0.122**	0.021	-0.004	**-0.055**	0.002	-0.028	-0.004	**-0.055**	**-0.085**	**-0.090**	**-0.090**	-0.030	**-0.060**	-0.030	**0.049**
siblings	-0.038	**-0.061**	0.030	**0.066**	0.013	-0.029	-0.004	**0.070**	-0.027	-0.047	-0.031	-0.029	**-0.065**	**-0.058**	**-0.048**	0.020
family situation	0.023	0.006	0.017	**-0.042**	-0.005	-0.056	0.005	-0.025	**-0.055**	-0.013	-0.003	-0.015	**-0.062**	-0.029	-0.002	-0.030
economic situation	-0.017	**-0.047**	0.026	**-0.074**	-0.010	-0.004	0.005	**-0.082**	**-0.108**	**-0.100**	-0.032	-0.032	**-0.069**	-0.021	-0.004	**-0.044**
drugs prescribed	**0.047**	-0.016	**0.050**	0.017	0.026	-0.026	-0.027	-0.008	-0.007	**0.078**	-0.015	-0.005	-0.040	**-0.095**	**-0.058**	**0.110**
drugs non-prescribed	**0.046**	0.037	-0.001	0.023	-0.008	**0.092**	**0.047**	**0.046**	0.012	0.017	**0.072**	0.035	0.008	-0.040	0.031	-0.010
practical doctor visits	0.028	0.002	0.012	0.031	0.003	**-0.071**	0.020	0.018	-0.006	0.040	0.025	**0.060**	0.027	-0.019	-0.013	0.039
antibiotics	0.005	0.016	-0.003	-0.004	-0.021	-0.039	**0.049**	-0.028	0.002	0.047	0.013	**0.051**	**0.062**	0.041	0.011	0.014
medical specialists visited	0.025	0.004	0.017	0.006	0.019	**-0.104**	**0.047**	0.000	**0.061**	0.038	0.028	**0.085**	0.033	0.003	-0.035	**0.054**
anxiety	0.007	0.041	-0.018	0.013	-0.014	-0.021	**0.056**	0.025	**0.049**	0.021	**0.055**	**0.069**	0.042	**0.051**	0.019	0.002
phobia	-0.005	**0.054**	**-0.046**	0.017	-0.040	0.012	**0.057**	0.015	**0.064**	0.017	-0.003	0.037	0.011	0.004	-0.024	**0.045**
depression	-0.012	**0.045**	-0.038	0.030	0.004	0.004	**0.095**	0.038	**0.066**	0.036	**0.076**	**0.084**	**0.072**	**0.087**	**0.044**	0.025
mania	-0.040	-0.001	-0.009	0.022	-0.013	0.008	**0.085**	-0.001	0.028	0.057	0.043	**0.076**	**0.086**	**0.062**	**0.067**	0.012
obsession	0.003	0.010	0.006	**0.046**	**0.050**	-0.071	**0.076**	-0.043	0.002	0.065	0.027	0.032	0.033	0.033	**0.067**	-0.016
audial hallucination	-0.042	0.012	-0.014	0.046	0.041	-0.009	**0.059**	**0.059**	**0.048**	0.047	**0.055**	**0.100**	0.043	0.022	**0.050**	0.008
visual halucination	-0.042	-0.006	-0.007	0.046	0.003	**0.077**	**0.075**	**0.093**	**0.069**	**0.103**	**0.055**	**0.121**	0.030	0.002	0.009	0.023
headache	-0.013	0.010	-0.022	**0.055**	-0.036	0.054	**0.045**	**0.048**	0.028	0.004	0.030	**0.085**	0.013	0.020	0.003	0.027
subjective physical health problems	-0.013	0.009	-0.033	-0.019	0.007	-0.015	0.002	**0.047**	0.038	0.032	-0.017	0.032	-0.002	**-0.096**	-0.025	**0.256**
subjective mental health problems	-0.028	-0.005	-0.014	0.013	-0.037	0.028	0.010	0.025	0.005	0.025	0.004	0.009	0.023	-0.001	0.001	**0.048**
diagnosed psychiatric disorders	0.017	**0.050**	-0.029	0.027	0.022	0.013	**0.061**	**0.059**	**0.102**	0.063	**0.107**	**0.097**	**0.055**	-0.037	-0.033	**0.058**
non-diagnosed psychiatric disorders	0.022	**0.061**	-0.036	**0.048**	0.011	-0.043	**0.050**	**0.056**	**0.058**	0.060	0.032	0.010	**0.057**	0.020	**0.070**	0.038
psychiatric disorders total number	0.022	**0.067**	-0.041	0.039	0.017	-0.049	**0.067**	**0.057**	**0.091**	**0.079**	**0.083**	**0.048**	**0.070**	0.004	0.037	**0.048**
partner's diagnosed psychiatric disorders	0.007	0.030	-0.017	0.026	0.003	-0.016	**0.044**	**0.047**	-0.023	0.035	0.019	-0.005	-0.041	-0.013	**0.050**	0.038
partner's non-diagnosed psychiatric disord.	-0.003	**0.044**	-0.035	0.009	0.023	-0.032	-0.027	0.034	0.003	0.009	0.015	-0.014	-0.002	-0.011	0.042	**0.063**
partner's psychiatric disord. total number	0.000	**0.055**	-0.038	0.021	0.001	-0.018	0.025	**0.046**	-0.001	0.015	0.030	0.000	-0.025	-0.007	**0.054**	**0.062**
mental health problems score	-0.012	**0.050**	-0.042	0.031	-0.001	-0.016	**0.090**	**0.053**	**0.082**	0.057	**0.086**	**0.103**	**0.062**	**0.056**	**0.051**	0.036
physical health problems score	**0.048**	0.030	0.011	0.027	0.000	-0.043	0.040	0.021	0.027	**0.093**	**0.051**	**0.074**	0.037	-0.034	-0.021	**0.060**
sexual activity	0.032	0.029	-0.002	0.036	0.030	-0.008	**0.061**	0.014	0.034	0.058	**0.058**	**0.093**	**0.245**	**0.125**	**0.125**	0.031
sexual desire	**0.090**	-0.019	**0.083**	**-0.064**	-0.001	0.045	-0.010	**-0.090**	**-0.071**	0.027	**-0.115**	**-0.092**	0.015	-0.025	0.016	-0.026

b) p-values of two-sided tests

	like dogs	like cats	prefer dogs	dog ever	dog now	dogs number	dog bit	cat ever	cat now	cats number	cat bit	cat scratched	smoking	alcohol	illegal drugs	BMI
WHOQOL-BREF health	0.360	0.830	0.438	0.268	0.249	0.013	0.058	0.007	0.236	0.538	0.110	0.007	0.524	0.050	0.331	0.000
WHOQOL-BREF psychological	0.400	0.518	0.835	0.846	0.225	0.030	0.070	0.515	0.255	0.978	0.042	0.134	0.081	0.086	0.593	0.007
WHOQOL-BREF social relationships	0.233	0.068	0.472	0.861	0.425	0.285	0.109	0.499	0.971	0.766	0.938	0.371	0.858	0.025	0.245	0.070
WHOQOL-BREF environment	0.761	0.918	0.359	0.569	0.629	0.025	0.112	0.018	0.000	0.342	0.248	0.133	0.143	0.080	0.554	0.212
WHOQOL-BREF total score	0.684	0.922	0.462	0.278	0.313	0.008	0.059	0.024	0.026	0.394	0.101	0.038	0.328	0.223	0.675	0.001
children	0.000	0.000	0.305	0.830	0.007	0.959	0.175	0.846	0.007	0.011	0.000	0.000	0.164	0.005	0.160	0.012
siblings	0.065	0.003	0.140	0.001	0.514	0.372	0.854	0.001	0.190	0.160	0.134	0.160	0.003	0.007	0.026	0.306
family situation	0.265	0.786	0.398	0.039	0.823	0.085	0.811	0.220	0.007	0.709	0.890	0.476	0.004	0.173	0.937	0.126
economic situation	0.398	0.021	0.199	0.000	0.612	0.892	0.812	0.000	0.000	0.003	0.123	0.116	0.001	0.326	0.847	0.025
drugs prescribed	0.029	0.459	0.020	0.442	0.233	0.447	0.203	0.704	0.729	0.026	0.478	0.819	0.062	0.000	0.007	0.000
drugs non-prescribed	0.035	0.087	0.982	0.294	0.700	0.008	0.030	0.035	0.588	0.630	0.001	0.104	0.705	0.065	0.156	0.650
practical doctor visits	0.202	0.937	0.575	0.158	0.892	0.041	0.356	0.398	0.774	0.252	0.257	0.006	0.206	0.388	0.539	0.075
antibiotics	0.814	0.463	0.892	0.849	0.336	0.262	0.025	0.190	0.937	0.183	0.552	0.020	0.004	0.058	0.613	0.528
medical specialists visited	0.250	0.859	0.446	0.777	0.390	0.003	0.029	0.982	0.005	0.284	0.197	0.000	0.131	0.890	0.103	0.013
anxiety	0.741	0.063	0.421	0.553	0.528	0.542	0.011	0.262	0.026	0.553	0.013	0.002	0.054	0.020	0.393	0.926
phobia	0.826	0.017	0.043	0.455	0.073	0.732	0.011	0.508	0.004	0.641	0.898	0.097	0.636	0.855	0.280	0.046
depression	0.584	0.045	0.093	0.184	0.859	0.916	0.000	0.089	0.003	0.329	0.001	0.000	0.001	0.000	0.049	0.272
mania	0.083	0.982	0.701	0.346	0.565	0.826	0.000	0.971	0.234	0.131	0.066	0.001	0.000	0.007	0.004	0.600
obsession	0.913	0.674	0.806	0.045	0.028	0.051	0.001	0.057	0.940	0.081	0.240	0.169	0.152	0.149	0.003	0.497
audial hallucination	0.075	0.621	0.538	0.051	0.082	0.807	0.012	0.012	0.040	0.223	0.020	0.000	0.070	0.357	0.034	0.722
visual halucination	0.074	0.812	0.780	0.054	0.886	0.043	0.002	0.000	0.004	0.008	0.021	0.000	0.209	0.933	0.704	0.333
headache	0.549	0.648	0.311	0.012	0.107	0.125	0.039	0.030	0.204	0.908	0.180	0.000	0.555	0.354	0.892	0.214
subjective physical health problems	0.561	0.674	0.147	0.395	0.763	0.679	0.917	0.037	0.088	0.377	0.454	0.155	0.937	0.000	0.265	0.000
subjective mental health problems	0.215	0.828	0.526	0.550	0.102	0.435	0.643	0.263	0.826	0.486	0.858	0.682	0.295	0.958	0.948	0.032
diagnosed psychiatric disorders	0.440	0.022	0.178	0.205	0.301	0.718	0.005	0.007	0.000	0.075	0.000	0.000	0.011	0.084	0.129	0.007
non-diagnosed psychiatric disorders	0.301	0.005	0.102	0.027	0.613	0.213	0.021	0.010	0.008	0.088	0.136	0.632	0.008	0.365	0.001	0.076
psychiatric disorders total number	0.314	0.002	0.058	0.072	0.428	0.156	0.002	0.009	0.000	0.025	0.000	0.027	0.001	0.838	0.084	0.028
partner's diagnosed psychiatric disorders	0.745	0.161	0.424	0.232	0.893	0.641	0.040	0.029	0.284	0.319	0.371	0.832	0.058	0.540	0.022	0.083
partner's non-diagnosed psychiatric disord.	0.887	0.042	0.112	0.666	0.288	0.350	0.206	0.113	0.902	0.790	0.499	0.519	0.918	0.614	0.052	0.003
partner's psychiatric disord. total number	0.982	0.011	0.078	0.337	0.965	0.598	0.253	0.032	0.964	0.668	0.163	0.987	0.250	0.745	0.013	0.004
mental health problems score	0.594	0.021	0.055	0.148	0.961	0.638	0.000	0.015	0.000	0.104	0.000	0.000	0.004	0.009	0.019	0.097
physical health problems score	0.028	0.161	0.597	0.210	0.983	0.218	0.062	0.342	0.219	0.008	0.019	0.001	0.087	0.114	0.337	0.005
sexual activity	0.165	0.218	0.942	0.117	0.200	0.832	0.008	0.534	0.139	0.119	0.013	0.000	0.000	0.000	0.000	0.188
sexual desire	0.000	0.413	0.000	0.007	0.979	0.238	0.674	0.000	0.003	0.476	0.000	0.000	0.532	0.292	0.505	0.274
