# Supplementary material for: Friends with malefit. The effects of keeping dogs and cats, sustaining animal-related injuries and Toxoplasma infection on health and quality of life
Source: PLoS One. 2019 Nov 22;14(11):e0221988. doi: 10.1371/journal.pone.0221988 (PMC6874301; doi:10.1371/journal.pone.0221988)
Supplement: S15 Table — (PDF) [file pone.0221988.s030.pdf]

Table S15: Hypothetical explanations of the observed associations

	**Positive association of liking pets with wellbeing**
1	Liking pets (or human subjects) has a positive effect on wellbeing.
2	People with good quality of life have a higher tendency to keep pets.
3	Optimistic people have a higher tendency to report liking pets as well as having a high quality of life.
	**Negative association of liking pets with health**
4	People with worse health have a higher tendency to like pets.
5	People who like pets have a tendency to keep pets, and keeping pets has a negative direct or indirect effect on their health.
6	Some third factor (e.g. the absence of social suport), has a negative effect on health and a positive effect on the probability of keeping pets.
	**Negative association of keeping pets with wellbeing and health**
7	Keeping pets has negative effects on health and, probably secondarily, on wellbeing.
8	Poor quality of life incites people to keep pets.
9	Keeping pets has a negative effect on wellbeing (e.g. by impairing the economic situation of a keeper), which could have a negative effect on health.
10	Some third factor (e.g. the absence of social suport), has a negative effects on quality of life and health and positive effects on the probability of keeping pets.
	**Negative association of sustaining pets-related injury with wellbeing and health**
11	Sustaining an injury has a direct negative effect on health (e.g. due to postraumatic stress disorder), and secondarily on wellbeing.
12	Sustaining an injury has an indirect (pathogens mediated) negative effect on health and secondarily on wellbeing.
13	Pets have higher a tendency to injure the people who are in worse (mental) health conditions; such people also have worse quality of life.
14	People in worse (mental) health conditions, who have also worse quality of life, have a higher tendency to report sustained injuries.
15	Pessimistic people have a higher probabability of reporting pet-realated injuries as well as other health and quality of life problems.
